# Hybrid Ubiquitous Coaching With a Novel Combination of Mobile and Holographic Conversational Agents Targeting Adherence to Home Exercises: Four Design and Evaluation Studies

**DOI:** 10.2196/23612

**Published:** 2021-02-22

**Authors:** Tobias Kowatsch, Kim-Morgaine Lohse, Valérie Erb, Leo Schittenhelm, Helen Galliker, Rea Lehner, Elaine M Huang

**Affiliations:** 1 Centre for Digital Health Interventions Department of Management, Technology, and Economics ETH Zurich Zurich Switzerland; 2 Centre for Digital Health Interventions Institute of Technology Management University of St Gallen St Gallen Switzerland; 3 Future Health Technologies Programme Campus for Research Excellence and Technological Enterprise Singapore-ETH Centre Singapore Singapore; 4 Graduate School of Culture Technology Korea Advanced Institute of Science and Technology Daejeon Republic of Korea; 5 People and Computing Lab Department of Informatics University of Zurich Zurich Switzerland

**Keywords:** ubiquitous coaching, augmented reality, health care, treatment adherence, design science research, physiotherapy, chronic back pain, pain, chronic pain, exercise, adherence, treatment, conversational agent, smartphone, mobile phone

## Abstract

**Background:**

Effective treatments for various conditions such as obesity, cardiac heart diseases, or low back pain require not only personal on-site coaching sessions by health care experts but also a significant amount of home exercises. However, nonadherence to home exercises is still a serious problem as it leads to increased costs due to prolonged treatments.

**Objective:**

To improve adherence to home exercises, we propose, implement, and assess the novel coaching concept of hybrid ubiquitous coaching (HUC). In HUC, health care experts are complemented by a conversational agent (CA) that delivers psychoeducation and personalized motivational messages via a smartphone, as well as real-time exercise support, monitoring, and feedback in a hands-free augmented reality environment.

**Methods:**

We applied HUC to the field of physiotherapy and conducted 4 design-and-evaluate loops with an interdisciplinary team to assess how HUC is perceived by patients and physiotherapists and whether HUC leads to treatment adherence. A first version of HUC was evaluated by 35 physiotherapy patients in a lab setting to identify patients’ perceptions of HUC. In addition, 11 physiotherapists were interviewed about HUC and assessed whether the CA could help them build up a working alliance with their patients. A second version was then tested by 15 patients in a within-subject experiment to identify the ability of HUC to address adherence and to build a working alliance between the patient and the CA. Finally, a 4-week n-of-1 trial was conducted with 1 patient to show one experience with HUC in depth and thereby potentially reveal real-world benefits and challenges.

**Results:**

Patients perceived HUC to be useful, easy to use, and enjoyable, preferred it to state-of-the-art approaches, and expressed their intentions to use it. Moreover, patients built a working alliance with the CA. Physiotherapists saw a relative advantage of HUC compared to current approaches but initially did not see the potential in terms of a working alliance, which changed after seeing the results of HUC in the field. Qualitative feedback from patients indicated that they enjoyed doing the exercise with an augmented reality–based CA and understood better how to do the exercise correctly with HUC. Moreover, physiotherapists highlighted that HUC would be helpful to use in the therapy process. The longitudinal field study resulted in an adherence rate of 92% (11/12 sessions; 330/360 repetitions; 33/36 sets) and a substantial increase in exercise accuracy during the 4 weeks.

**Conclusions:**

The overall positive assessments from both patients and health care experts suggest that HUC is a promising tool to be applied in various disorders with a relevant set of home exercises. Future research, however, must implement a variety of exercises and test HUC with patients suffering from different disorders.

## Introduction

Musculoskeletal disorders (MSDs), such as rheumatoid arthritis, osteoarthritis, low back pain, neck pain, or gout, negatively impact the locomotor system and are influenced by various risk factors, such as a sedentary lifestyle, malnutrition, or obesity [1]. Individuals with MSDs suffer from chronic pain, impaired mobility and physical function, and reduced quality of life [2]. The average estimated global prevalence of MSDs lies at 18% [3] and increases with age [4]. MSDs account for 21.3% of all years lived with disability worldwide [5], with back pain as the leading cause of disability [3]. Therefore, both the individual’s psychosocial burden and the financial burden of MSDs are significant. In the United States, for example, treatment costs are estimated to account for 5.7% of gross domestic product [6]. As a consequence of population growth, aging, and sedentary lifestyles, the burden of MSDs will dramatically increase in the future and thus poses significant challenges to global health [4,5]. It is, therefore, crucial to develop effective interventions for individuals with MSDs.

Physiotherapy is an effective intervention for MSDs [7-10]. However, treatment adherence (ie, the extent to which an individual’s behavior “corresponds with agreed recommendations from a health care provider” [11]) is a common problem in various health care settings [11-13], including home exercises in physiotherapy [11,14-17]. For example, treatment adherence ranges from 60% [18] down to 30% [9,19,20], resulting in increased costs because of prolonged treatment [5,8,21-23]. Common treatment adherence dimensions (TADs) [24-26] are the session completion rate (the number of completed vs prescribed exercise sessions, TAD1), the set completion rate (the number of completed vs prescribed sets for each exercise, TAD2), the exercise repetition rate (the number of completed vs prescribed exercise repetitions within each set, TAD3), the temporal exercise accuracy (the actual vs prescribed velocity of exercise execution, TAD4), and the spatial exercise accuracy (the actual vs prescribed body movement trajectory, TAD5). Reasons for nonadherence can vary. For example, patients may simply forget to follow the various TADs, and further patient-related factors, such as disability, attitudes, motivation, and beliefs about exercise risks and benefits, can also negatively impact the TADs [14,27-33].

Moreover, adherence support is often limited to on-site physiotherapy sessions, leaving patients alone at home with standardized exercise instructions, which results in low adherence rates [34-36]. Therefore, various technical approaches have been proposed to help increase home exercise adherence in physiotherapy [37]. A smartphone app, for example, motivates patients and sends reminder messages to individuals with an MSD [38], or a virtual reality training is used to provide feedback on exercise execution [39]. However, two recent reviews indicate that adherence remains a challenge [40,41]. Our review of remote patient monitoring tools and current scientific work (Multimedia Appendix 1 [16,29,39,42-66]) suggests that there is untapped potential in addressing this problem, too.

**Figure 1:**
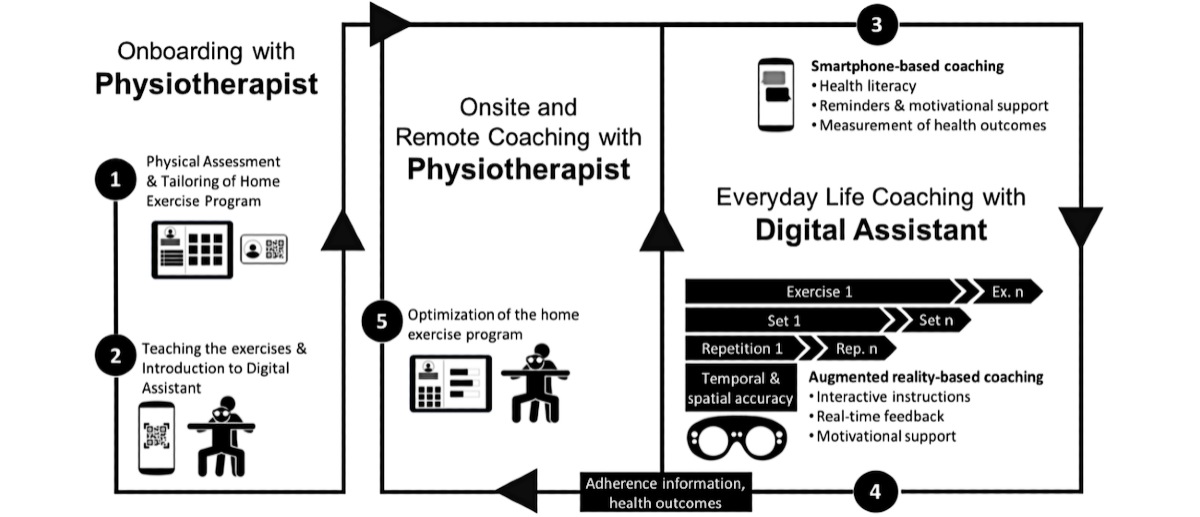
Overview of hybrid ubiquitous coaching (HUC).

To this end, we propose hybrid ubiquitous coaching (HUC) to improve home exercise adherence for treatments that require not only personal on-site coaching sessions by health care experts but also a significant amount of home exercises. HUC relies primarily on a conversational agent (CA) that delivers relevant health literacy information about the importance and benefits of home exercises and personalized motivational exercise reminders via a smartphone. Moreover, the CA of HUC delivers real-time exercise support, monitoring, and feedback in a hands-free augmented reality (AR) environment. In HUC, health care experts introduce the CA as their trusted personal assistant that lives not only in the patient’s pocket/smartphone in the form of a text-based physiotherapy chatbot [48,49,67], but also in the patient’s AR glasses in the form of an embodied, holographic instructor [52,55] (see Figure 1 for an overview of HUC).

HUC combines for the very first time (to the authors’ best knowledge) research on CAs, human-supported digital interventions, health psychology, and mobile and wearable technology–supported physical exercises. First, HUC employs a CA because they are able to build a working alliance with patients [49,57,68], which is an important relationship quality [69] that is robustly linked to treatment success [59]. Accordingly, there is a large body of evidence on the effectiveness of CAs in delivering clinical and nonclinical interventions [52]. Second, HUC relies on a hybrid coaching concept consisting of CA and human physiotherapist teams because digital interventions supported by humans result not only in higher treatment adherence [64,66], but also in better treatment outcomes [70,71]. Third, HUC offers real-time feedback about intervention progress and correct exercise execution, which, in turn, is assumed to increase self-efficacy [29], an important construct that helps shape positive attitudes toward therapy adherence and health-promoting behavior [63]. Finally, HUC aims to increase adherence by seamlessly delivering an intervention into the everyday lives of vulnerable individuals or patients with the help of mobile and wearable technology (ie, smartphones and AR glasses) [72,73].

Because this research is unprecedented, the following research questions (RQs) were formulated to guide the design, implementation, and evaluation of HUC: (RQ1) How is HUC perceived by (a) patients and (b) health care experts?; and (RQ2) Does HUC lead to treatment adherence to home exercises?

To answer these questions, we applied HUC exemplarily to the field of physiotherapy. During the last 3 years, 4 design-and-evaluate loops were conducted: two studies were carried out in the lab with 50 physiotherapy patients, 11 physiotherapists were interviewed, and finally, empirical data from 1 patient during a 4-week longitudinal field study were collected and assessed to reveal real-world benefits and challenges related to HUC.

## Methods

### Overview

To address the challenge of nonadherence to home exercises in physiotherapy, we started the interdisciplinary development of HUC in collaboration with 2 physiotherapists. In a first step, a storybook was developed (Multimedia Appendix 2). In a second step, HUC was developed further as outlined in the next section. After the conceptual work, we formulated the research questions that guided the 4 studies in which we iteratively improved, implemented, and evaluated HUC. Table 1 provides an overview of the 2-year design and evaluation process.

**Table 1:** Overview of the studies.

Characteristic	Study 1, 2018	Study 2, 2018/2019	Study 3, 2019		Study 4, 2019
Maturity of HUC^a^	Version 1: AR^b^-based CA^c^ coaching. Superhero exercise instructor and humanlike female exercise model.	Version 2: Revised AR-based CA coaching. One humanlike exercise instructor with professional physiotherapist clothing and additional smartphone-based CA coaching (mockup).		Version 3: Revised AR-based CA with dynamic behavior over time and Wizard of Oz smartphone-based CA coaching	
Study design	Lab study	Semistructured interview and survey	Within-subject lab study		Observational longitudinal field study
Participants	35 patients	11 physiotherapists	15 patients, 2 physiotherapists		1 patient-physiotherapist dyad, 3 physiotherapists
RQ1a: How is HUC perceived by patients?	Perceived usefulness, ease of use & enjoyment.^d^ Suggestions for improvements.^e^	N/A^f^	Patient-CA working alliance.^d^ Perceived usefulness, ease of use, enjoyment, task load, exercise difficulty.^d,e^		Perceived usefulness, ease of use & enjoyment. Intention to continue using HUC, suggestions for improvements.^d^
RQ1b: How is HUC perceived by health care experts?	N/A	Patient-physiotherapist working alliance, relative advantage of HUC.^d,e^	N/A		Perceived usefulness and relative advantage of HUC, suggestions for improvements.^e^
RQ2: Does HUC lead to treatment adherence to home exercises?	Behavioral intention & recommendation to use HUC.^e^	Session completion rate, sets & repetition (TAD1-3^g^)^d^	Spatial & temporal accuracy (TAD4-5)^d^		Session and set completion rates, exercise repetition rate, spatial & temporal accuracy (TAD1-5)^d^

^a^HUC: hybrid ubiquitous coaching.

^b^AR: augmented reality.

^c^CA: conversational agent.

^d^Quantitative feedback.

^e^Qualitative feedback.

^f^N/A: not applicable.

^g^TAD: treatment adherence dimension.

### Concept of Hybrid Ubiquitous Coaching

A conceptual overview of HUC is illustrated in Figure 1 and outlined in the following paragraphs.

#### Onboarding With a Physiotherapist

First, a physiotherapist performs a physical assessment of the patient and, depending on the results, defines a tailored home exercise program. Next, the physiotherapist works together with the patient on the exercises from the program by focusing on the TADs (Table 1). Then, the physiotherapist introduces the CA as his or her scalable personal assistant that will support the patient regarding the TADs in their everyday life via the smartphone and the AR glasses. For this purpose, the physiotherapist shows the patient how to interact with the CA on the smartphone and via the AR glasses. To link the CA with the patient (eg, name, age, height, weight, diagnosis) and his or her specific home exercise program, the physiotherapist prints out a therapeutic prescription card with a quick response (QR) code. The QR code not only contains encrypted links to the patient data and personalized exercise program, but also data about the physiotherapist so that the CA “knows” the name of the physiotherapist it was linked to. This allows the CA to link back to the “real” world during conversations with the patient and strengthen the relationship and the working alliance with the specific physiotherapist. A strong working alliance between health professionals and patients establishes an attachment bond [74], the development of a shared understanding about goals and tasks, and, in return, boosts treatment adherence [69,75]. On the way home, the patient scans the QR code with his or her smartphone to download the mobile HUC app and start the interaction with the CA. The CA introduces itself as the physiotherapist’s personal assistant and gives an overview of the upcoming digital coaching program. During this dialogue with the CA, the shipment of the AR hardware to the patient’s home is triggered as the patient’s address is made available via the link in the QR code.

#### Everyday Coaching

The smartphone- and AR-based CAs allow for remote everyday coaching. The combination of the two CAs together results in a richer communication channel that enables more accurate and personalized information to be transmitted to the patient [76,77]. The design of the HUC elements was also informed by behavioral change techniques (BCTs, ie, evidence-based intervention components aiming to change behavior) [78]. The smartphone-based CA reminds the patient to do the home exercises (TAD1), motivates the patient via the smartphone, and emphasizes the benefits of performing the exercise by providing psychoeducational material, for example, about sleep quality and degree of pain (BCTs 2, 13, 15, and 20 [78]). The AR-based CA delivers real-time exercise support by demonstrating the execution (BCT 22), whereby arrows highlight important angles to be aware of and motivate the user during the exercise (BCT 13). The AR-based CA guides the patient through the exercise, monitoring the progress (TAD1-5, BCT 17) and giving real-time feedback (TAD4-5, BCT 19). It also informs and emphasizes essential aspects of how to perform the exercise correctly (TAD4-5, BCT 21). The AR-based CA counts the number of sets and repetitions out loud and provides feedback on the exercise execution after a completed set of exercises (TAD2-5, BCT 19). The feedback is both visual and auditory and is based on comparing patient data from the AR system with data about the physiotherapist performing the same exercise. Lastly, the AR-based CA aims at increasing the attachment bond, an important relationship quality and dimension of a working alliance [79], by literally giving high fives or showing the patient a funny dance move (BCT 13) when a set or training session is completed.

#### On-site and Remote Coaching With a Physiotherapist

HUC grants physiotherapists access to patients’ data about the TADs via a web-based dashboard. Based on that data, physiotherapists can tailor and optimize planned on-site and remote coaching sessions more efficiently. For example, if squats are performed incorrectly and the real-time feedback from the AR-based CA is not processed correctly by patients, pressure could be applied on the knees rather than on the muscles, which could cause knee injuries. If this happens, the physiotherapist would get an alert message from the CA to target the weakness directly in the next online or on-site coaching session.

In the remainder of this paper, we describe 4 build-and-evaluate loops that we conducted to build and assess HUC according to our research questions. Table 1 provides an overview of this process.

### Study 1: Perceptions of Individuals With Physiotherapy Experience

To address RQ1a and RQ2, we implemented a first version of HUC with a focus on the AR-based CA and tested it with 35 individuals with physiotherapy experience at a public fair in September 2018.

#### Development of HUC Version 1

**Figure 2:**
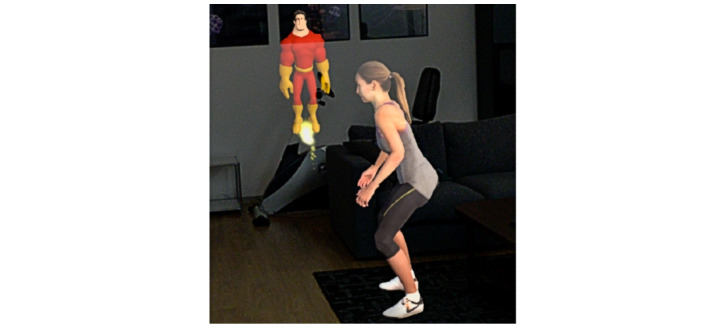
Augmented reality–based conversational agent and female humanlike model demonstrating the squat exercise.

**Figure 3:**
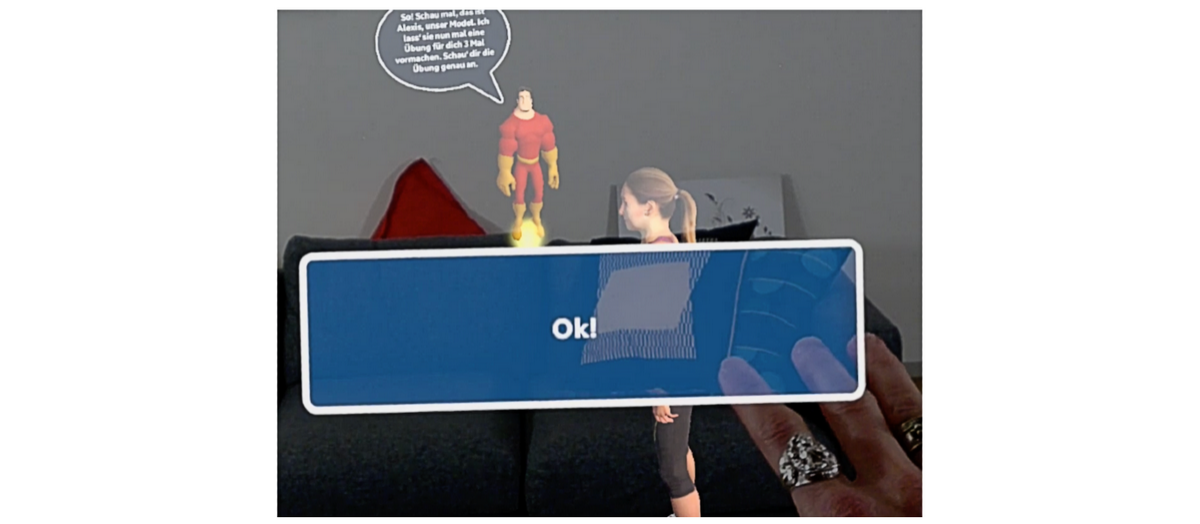
Predefined answer options. The conversational agent communicated visually and auditorily.

**Figure 4:**
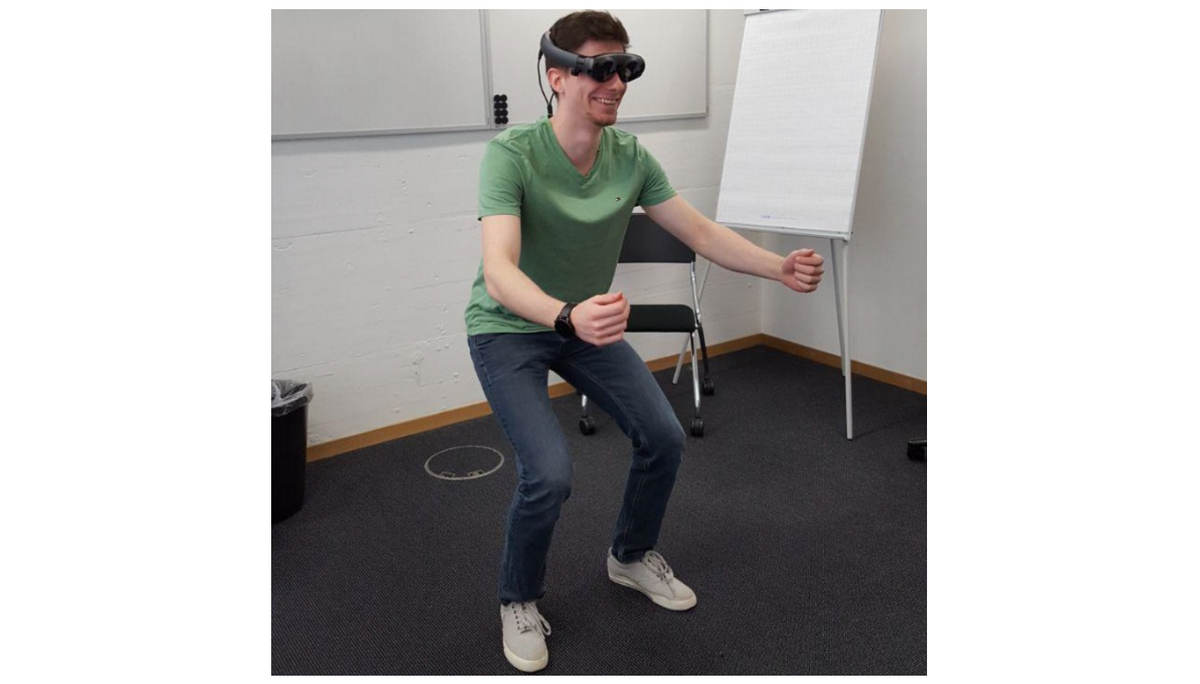
Participant performing the squat exercise while wearing the augmented reality hardware.

HUC included an AR-based CA that appeared as a male cartoonlike superhero assistant of a human male physiotherapist and a female human-looking exercise model, demonstrating the exercise execution (Figure 2). A personal, empathetic, and humanlike CA can increase a patient’s intention to change behavior and to continue to use the CA [80]. Further, the rationale behind the superhero design was to make the user experience more fun. The rationale for the design features was to design a personal and empathetic CA. For ease of presentation, a squat exercise was implemented as it is a common physiotherapy exercise. After the CA demonstrated the exercise (eg, by flying around the female character and pointing out important aspects of the squat movements) and commented via voice on how to correctly execute the squat, the CA asked the patient to perform the exercise. Patients were able to respond to voice-based questions from the CA with predefined answer options that appeared as speech bubbles in the AR space around 20 cm in front of and at eye level for the patient (Figure 3). The patient was able to select an answer by touching it with a finger. This hands-free approach does not require an additional controller and thus allows for more intuitive and direct interactions [81] (Figure 4). A significant advantage of this is the direct manipulations and interactions in the AR space. Interactions were primarily used to increase the attachment bond via small talk (eg, “How are you today?”), to explain the exercise (eg, “Let’s have a close look at Alexis’ movements”), or to progress through the several-step process of exercise execution (“well done, four more to go!”). See Multimedia Appendix 3 for the video clip and Multimedia Appendix 4 for the technical details of the implementation and hardware.

#### Evaluation

##### Participant Acquisition

Participants were recruited from a public fair using convenience sampling [82]. They were included if they had already participated in physiotherapy sessions and were interested in HUC.

##### Procedure

Participants who expressed an interest in HUC and provided their consent to participate in the study were invited to participate. HUC was then introduced, and usage of the AR glasses was demonstrated. Next, participants were asked to start the interaction with the AR-based CA and performed one exercise session. Afterward, participants were asked to fill out a feasibility questionnaire. Participants received no monetary compensation.

##### Measurements

HUC was assessed based on technology acceptance [83-85] and word-of-mouth [86] research. To reduce participant burden, and due to the feasibility character of this first study, we used single-item measures for perceived enjoyment [87], perceived ease of use, perceived usefulness [85], and intention to use [83]. Consistent with prior work [87], 7-point Likert scales were used, ranging from strongly disagree (1) to strongly agree (7). Further, the Net Promoter Score (NPS) [86] was used to assess whether participants would recommend HUC to other patients. The NPS is a single-item measurement that indicates satisfaction with a service. NPS scores are binned into four categories: −100 to 0 (needs improvement), 1 to 30 (good), 31 to 70 (great), and 71 to 100 (excellent). Finally, qualitative feedback was gathered on positive aspects of HUC, and suggestions for improvement were provided by participants.

### Study 2: Revision of HUC and Evaluation by Physiotherapists

To address RQ1b and RQ2, we revised HUC based on the feedback from study 1 and assessed it with 11 physiotherapists between November 2018 and March 2019.

#### Design and Technical Implementation

**Figure 5:**
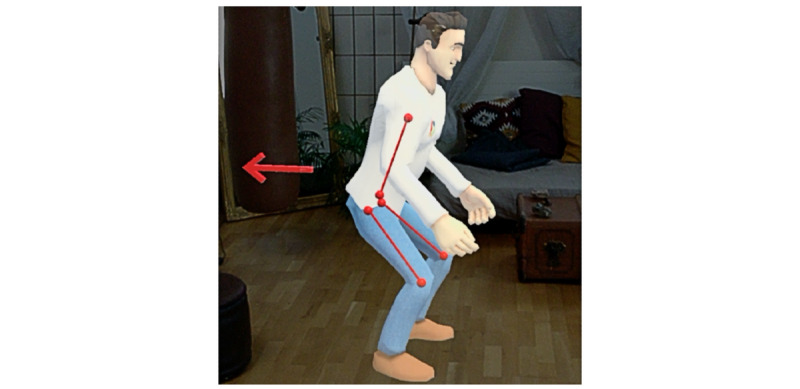
Virtual guides embedded into the revised augmented reality–based conversational agent and used in studies 2, 3, and 4.

HUC was revised as follows based on the qualitative results of study 1. The AR-based CA was scaled up to be human-sized and adapted in appearance to look like a physiotherapist (ie, the clothes, including the logo, were copied from a real physiotherapist, since a professional look and a natural humanlike style are agent characteristics associated with increased intention to use [80]). Moreover, due to the limited field of view of the AR glasses, which sometimes made it hard to see the interactions between the two characters in HUC version 1 and thus confused some of the participants in study 1, the female humanlike model was omitted. Therefore, in study 2 the revised AR-based CA demonstrated the squat exercise with the help of his own virtual body (Figure 5 and the video in Multimedia Appendix 5). Moreover, an exercise tutorial was added to help participants better understand how to correctly execute the exercise (BCT 22). For this purpose, virtual guides indicated important aspects of exercise execution (Figure 5). Furthermore, automatic error detection was implemented in collaboration with 2 physiotherapists. For the error detection, the position and rotation of the headset were tracked and compared with the squat movements of the physiotherapists (Multimedia Appendix 4). Participants were asked to position themselves on virtual footprints placed on the floor and to remain in that position during the course of the exercise. Thus, participants’ initial position and rotation were determined. Additionally, participants’ heights were stored as the difference between the initial position and the floor along the vertical axis. Based on these parameters, errors for insufficient depth of the squat, too fast or too slow execution, and too much deviation to the left, right, front, or back from an individual’s vertical center axis were detected. Further technical details of the error detection are outlined in Multimedia Appendix 4. The errors detected were saved in the AR app so that the CA would be able to generate and send an error report to the corresponding physiotherapist. For example, if a participant did not move low enough with the upper body during a specific squat exercise, the AR-based CA would say, “A little bit lower.” In addition to this error-related real-time feedback, and to target TAD1-3, the AR-based CA took over the moderation during the exercises with motivating voice-based instructions and progress reports (BCTs 12, 13, and 15). For example, the AR-based CA counted up the number of repetitions (eg, “Only three… two… one. Great! You already finished the 1st set, take a quick break and then let’s start with the 2nd set of squats”). Moreover, real-time feedback was implemented based on these errors and communicated during and after a specific exercise, thus targeting temporal (TAD4) and spatial (TAD5) accuracies.

Finally, the HUC concept outlined in Figure 1 was broken down into a flowchart diagram and specific sketches (eg, of the web-based dashboard for physiotherapists and the smartphone-based CA interaction; Multimedia Appendix 6) that illustrated the various intervention components of HUC in more detail. In addition to the revision of the AR-based exercise, the flowchart and sketches were then used for the assessment of the various aspects of HUC by physiotherapists as outlined below.

#### Evaluation

##### Participant Acquisition

Physiotherapists treating patients with MSDs were recruited using chain sampling [82] until saturation of the qualitative feedback was reached [88].

##### Procedure

First, we explained the HUC concept, the flowchart, and the sketches to each participating physiotherapist. Then, the physiotherapist performed the specific squat exercise session with the AR-based CA. Thereafter, a semistructured interview was conducted to gather feedback on the utility and feasibility of HUC. The interview was recorded and transcribed according to the rules of simple transcription [89]. Thereafter, relevant themes and concepts were extracted following an iterative coding process [90]. After the interview, we sent an online survey to the physiotherapist in which we asked them to assess the relative advantage of HUC compared to current patient monitoring and communication applications and asked about the potential of HUC to strengthen the working alliance between the physiotherapist and their patient. No monetary compensation was provided.

##### Measurements

The guiding questions of the semistructured interview are listed in Multimedia Appendix 7. The perceived relative advantage of HUC was adapted from prior work [91]. The answer options on the 6-item instrument were anchored on 7-point Likert scales, ranging from strongly disagree (1) to strongly agree (7). The Session Alliance Inventory [92] was used to assess the working alliance. Answer options on the 6-item instrument were anchored on 7-point Likert scales, ranging from never (1) to always (7).

### Study 3: Revised HUC (Version 2) Assessed by Patients

To address RQ1a and RQ2, HUC was assessed by 15 patients seeking physiotherapy treatment in January and February 2019. To assess the relative advantage of HUC to commonly employed methods of exercise instruction, HUC was compared to paper-based and video-based exercise instruction.

#### Design and Technical Implementation

The revised HUC (version 2) as outlined in study 2 above was used for the assessment.

#### Evaluation

##### Participant Acquisition

Patients were recruited from a physiotherapy center of one of the collaborating physiotherapists. Inclusion criteria were age ≥18, participation in at least 3 physiotherapy sessions, no experience with the squat exercise, being in the physical condition to perform squat exercises, normal vision or wearing contact lenses, and normal hearing.

##### Procedure

**Figure 6:**
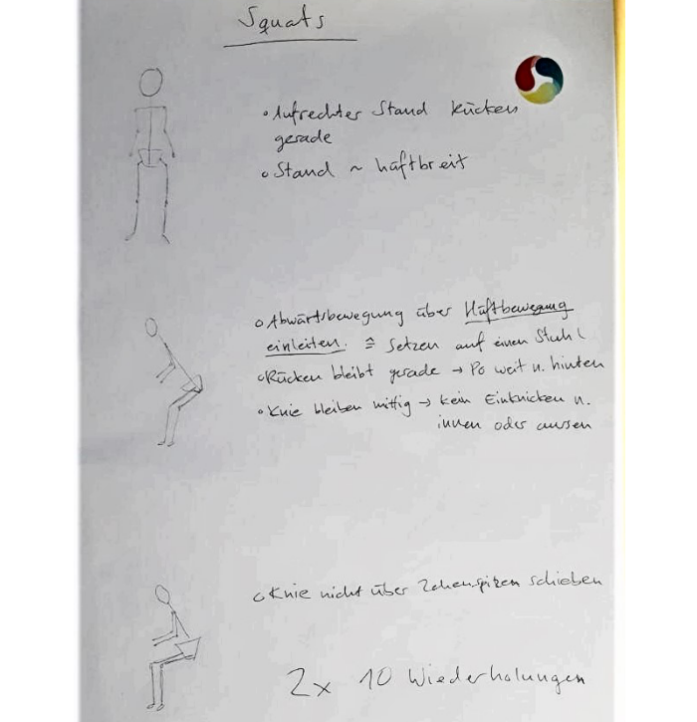
Paper-based instructions of study 3.

**Figure 7:**
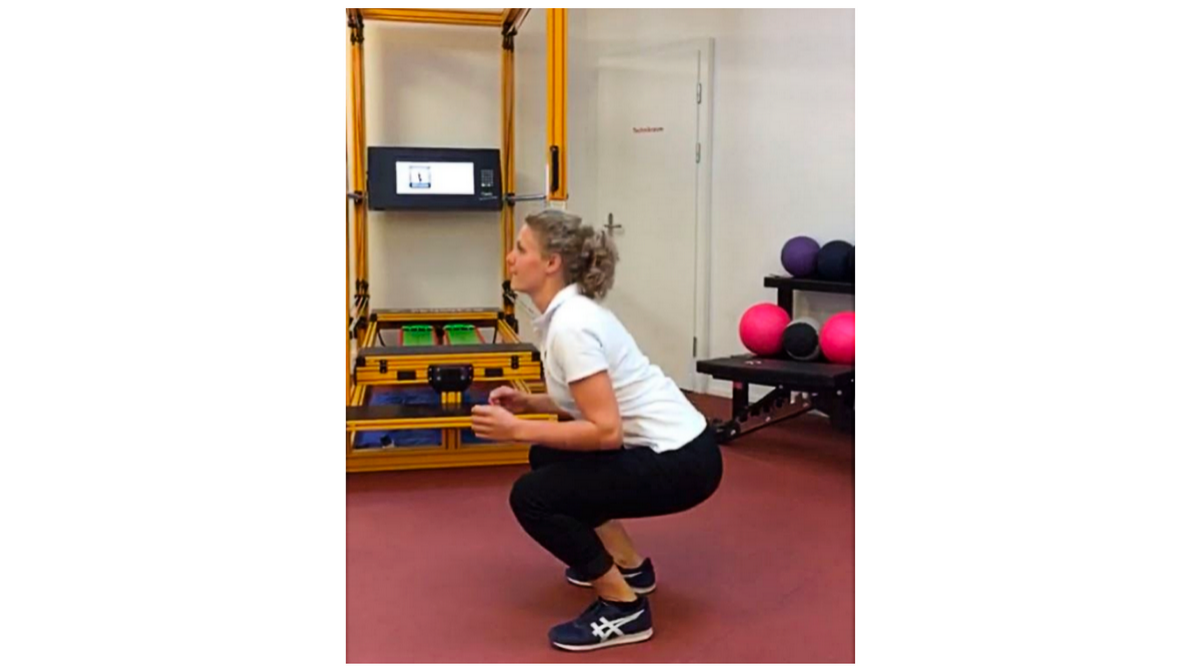
Video-based instructions of study 3.

A two-period crossover study design was used to assess the relative advantage of HUC compared to paper-based and video-based exercise instruction, two commonly used methods in physiotherapy. This study design also allowed us to account for learning effects [93]. The study was conducted at the physiotherapist center. After giving consent, each participant was systematically assigned to a specific order of the exercise instruction method. Then, a physiotherapist described and demonstrated the squat exercise in the therapy session and ensured correct exercise execution. Next, patients received either handwritten paper-based exercise instructions (Figure 6), video-based instructions (Figure 7 and Multimedia Appendix 8), or a QR code on a business card to download the HUC app and the AR hardware (Figure 5 and Multimedia Appendix 5). The content of the exercise instruction was consistent for all three instruction methods. Each patient was then instructed to perform 3 sets of squats with 10 repetitions each. The exercises were video-recorded so that 2 physiotherapists were able to assess the accuracy of the exercise execution independently from each other at a later point in time (see Measurements). After each of the 3 exercise sessions, patients were asked to assess the instruction method via an online survey. Finally (ie, after all exercise sessions), a semistructured interview was conducted with each participant to gather suggestions to improve HUC. The overall duration of approximately 1 hour was compensated with US $50.

##### Measurements

The exercise instructions were assessed with mental effort and frustration items from the task load index [94]. Moreover, perceived enjoyment, ease of use, and usefulness of the exercise instructions were adopted from technology acceptance research [85,95]. We also assessed the working alliance between the patient and HUC’s AR-based CA with the Session Alliance Inventory [92]. Additionally, the intention to continue working with the corresponding exercise instruction was determined based on prior work [96]. We also asked patients to rank the three instruction exercises according to their informativeness. We finally adopted 5 assessment items from the Exercise Assessment Scale [97]. With these items, 2 independent physiotherapists were asked to assess the correctness of the exercise execution for each instruction method and participant with a maximum score of 6 points. For this purpose, patients performing the squat were video-recorded from two perspectives (front and side). All quantitative survey items are listed in Multimedia Appendix 9. The questions of the semistructured interview focused on patients’ perceptions and suggestions for improvements (Multimedia Appendix 10).

### Study 4: HUC Assessed in a 4-Week Field Study

Due to the high burden nature of the study, with experimental technology that required support from the researchers, we conducted a 4-week n-of-1 trial to complement our findings from studies 1-3 and to answer RQ1a, RQ1b, and RQ2. The goal of this study was to complement the findings from the previous studies and to add external validity to the lab results by assessing the long-term adherence and feasibility of HUC in the everyday life of one patient. By assessing HUC with only one patient, this study was not designed to illustrate the breadth of possible use experiences but to show one experience in depth and thereby potentially reveal real-world benefits and challenges [98,99].

#### Design and Technical Implementation

The objective of the third design loop was to adapt HUC for long-term use. For the field study assessment of HUC, there was no QR code provided, and no dedicated HUC was developed. Consistent with recent work [80,100,101], the following revisions were implemented to increase variation in HUC coaching.

First, depending on the patient’s familiarity with the AR-based app (eg, how often it was used), tutorials became skippable, and explanations were shortened after the first week. Second, there was randomization of the coach’s speaking texts (“Hey, good to see you again!”), animations (eg, stretching the upper limb), and interactions (eg, clapping, high five, thumbs up). Third, randomization of the coach’s texture (ie, the AR-based CA had a set of different colored clothes) and the background music (eg, energetic rock and funk) changed. Lastly, the AR-based app showed the patient the number of sessions already completed as an early form of progress visualization, a well-established behavioral change technique (BCT 19) [78].

#### Evaluation

##### Participant Acquisition

A new physiotherapy patient who did not participate in one of the other studies was recruited from a physiotherapy center. The eligibility criteria were identical to those outlined in study 3. Additionally, 3 physiotherapists from the same center were recruited for a follow-up interview. One physiotherapist was the therapist treating the patient, the second therapist was involved in the evaluation in the course of the third study, and the third therapist was not involved in any of the previous studies.

##### Procedure

Before starting the study, the patient signed the informed consent. Then, at the beginning of the study, the patient received a study information sheet from the physiotherapist and agreed to practice a squat exercise 3 times per week. HUC was then demonstrated, and the patient received an introduction to the AR headset. Thereafter, the patient was coached by HUC in their everyday life as outlined in Figure 1. Again, the exercise in focus was the squat. The coaching included, on average, 4 weekly text messages via WhatsApp, aimed to elicit reminders, to provide psychoeducational material, and to motivate the patient to perform the squat as often as the physiotherapists recommended (BCTs 2, 13, 15, and 20 [78]). According to the Wizard-of-Oz method [102], the CA was simulated by a coauthor without the patient being aware of it and reminded the patient weekly to conduct the exercises over a 4-week period. Further, real-time feedback was provided during the execution of the exercises (Multimedia Appendix 5). The TAD data (eg, session completion rate, sets and repetitions, errors) were collected and sent to a coauthor via email so that additional feedback could be provided via a WhatsApp-based text message (eg, to motivate the patient to perform an exercise). After the trial, the patient was invited to take part in a debriefing interview, during which the experience with HUC was explored and the patient was asked for suggestions for improvement. The patient received a monetary compensation of US $50 for participating. In the case of any therapeutic or technical questions, the patient was able to contact the physiotherapist and the study team. Finally, the results of the n-of-1 trial were shown to 3 physiotherapists to gather additional qualitative feedback about HUC (eg, suggestions for improvement).

## Results

The results of the 4 studies are presented in the following sections. The depersonalized raw data and data analysis script are made available in Multimedia Appendices 9 and 11, respectively.

### Study 1: Perceptions of Individuals With Physiotherapy Experience

Overall, 35 (13 females) individuals with a mean age of 35 years (SD 11) and previous physiotherapy experience participated in the study. Most of them (32, 91%) had experience with the exercise. The results listed in Table 2 indicate that participants enjoyed the exercise sessions and perceived the exercise moderated by the AR-based CA to be useful and easy to use. Moreover, participants indicated that they would be willing to use this form of AR-based exercise at home. All of these assessments lie significantly above the neutral scale value by conducting Wilcoxon signed rank tests (Table 2). The NPS was negative and close to zero, indicating that the HUC needs improvement before participants would recommend it to other patients. This result was expected given the prototype character of this first version of HUC.

**Table 2:** Augmented reality–based conversational agent coaching assessed by 35 patients.

Construct	Items	Mean^a^ (SD)	*P* value^b^
Perceived enjoyment	I enjoyed the exercise with Alex.^c^	5.74 (1.06)	<.001
Perceived ease of use	It was easy to follow the exercise instructions.	6.14 (0.84)	<.001
Perceived usefulness	I was able to follow the exercise.	6.37 (0.80)	<.001
Intention to use	I would use this type of holographic exercise at home.	5.40 (1.49)	<.001
Net Promoter Score^d^	How likely is it that you would recommend this type of exercise to other patients?	−17.14^e^	N/A^f^

^a^7-point Likert scales ranging from strongly disagree (1) to strongly agree (7).

^b^Wilcoxon signed rank test with test value 4.

^c^The augmented reality–based conversational agent was given the name Alex.

^d^NPS ranges between −100 and 100.

^e^The percentage of detractors subtracted from the percentage of promoters.

^f^N/A: not applicable.

**Figure 8:**
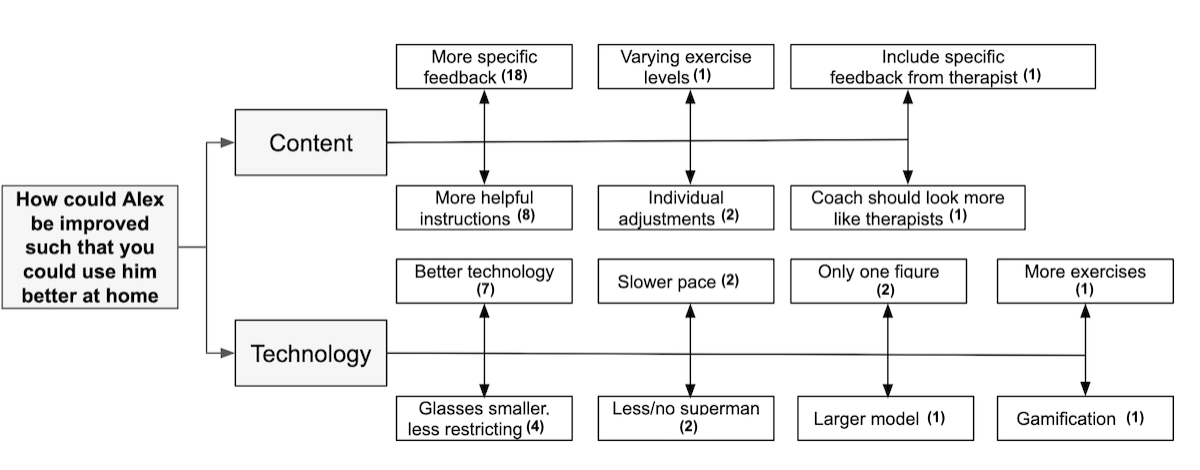
Thematic map of the patients' qualitative feedback of study 1. Note: the number in brackets indicates the frequency a topic was mentioned.

The qualitative feedback on the positive aspects of HUC and suggestions for improvement are shown in the thematic maps (Figure 8). Suggested improvements included “more specific feedback,” “more helpful instructions,” “coach should look more like a physiotherapist,” “only one figure,” and “less/no superman.”

### Study 2: Evaluation by Physiotherapists

Eleven physiotherapists with 3 to 30 years of experience (6 females, age of both sexes between 20 and 49 years) participated in the study. Five physiotherapists worked in a private practice, 4 in a hospital, and 2 in a rehabilitation clinic.

The physiotherapists indicated in the interviews that patients could be better supported with the HUC and that physiotherapists would intend to use it for treatment sessions. The AR-based CA was perceived as an additional motivator due to its personal, interactive, and playful approach. The exercise guidance by the life-sized CA, including the real-time feedback (eg, automatic counting of exercise repetitions), was also perceived as an improvement to the status quo. The smartphone-based CA was perceived as an advantage over current solutions, mainly due to its ability to remind patients to execute the exercise, but also by providing psychoeducational content that targets increasing patients’ health literacy. Physiotherapists were, however, skeptical of whether HUC could improve the quality of the treatment and adherence. The HUC was seen to have some potential to strengthen the working alliance between physiotherapists and their patients. Establishing therapeutic goals was seen as an advantage, since tracking enables patients to be continuously informed about the degree of goal achievement. The ability to instruct and provide feedback on the exercise technique was also identified as a factor that could potentially improve the shared understanding of treatment tasks. However, the majority of the physiotherapists expressed the belief that mutual trust, empathy, and, consequently, mutual goals and tasks were uniquely established during face-to-face encounters and that technological systems could not successfully act as assistants to foster a good working alliance. Lastly, physiotherapists suggested that continuously monitoring a patient could have a negative influence on mutual trust.

This qualitative feedback is supported by the quantitative data from the online survey (Table 3). The physiotherapists saw a clear relative advantage of HUC relative to the status quo of commercial applications. The working alliance assessments also resulted in a confirmation of the qualitative feedback. Overall, the experts assessed the support of the CA as not being sufficient to build and maintain a robust working alliance between patients and physiotherapists. This was especially the case for establishing an attachment bond.

**Table 3:** HUC assessed relative to commercial applications by 11 physiotherapists.

Construct		Exemplary item	Items, n	Mean^a^ (SD)	*P* value
Relative advantage		With HUC^b^, patients would perform the exercises more correctly compared to previous methods.	6	4.97 (0.96)	<.001^c^
**Working alliance**					
	Goal agreement	Alex would help me (my patient and I) work towards mutually agreed goals.	2	4.23 (1.31)	.19^d^
	Task agreement	Alex would help me convince the patient that the way we work on the problem is correct.	1	3.82 (1.47)	.64^d^
	Attachment bond	Alex would help me make the patient appreciate me.	3	2.06 (1.32)	.99^d^
	Total		6	3.08 (1.68)	.99^d^

^a^7-point Likert scales ranging from strongly disagree (1) to strongly agree (7) were used.

^b^HUC: hybrid ubiquitous coaching.

^c^*t* test (2-tailed; t_10_=8.204) with test value 4.

^d^Wilcoxon signed rank test with test value 4.

### Study 3: Revised HUC (Version 2) Assessed by Patients

Fifteen patients (9 female) with a mean age of 37 years (SD 9.93) participated in the study. Participants were generally very positive toward HUC, the video instruction, and, to some extent, the paper instructions (Table 4). In total, 8 patients (53%) rated HUC as their favorite training method, while 6 patients (40%) rated the video instructions as their favorite method. Further, in the HUC method, the overall working alliance, task agreement, and attachment bond values were statistically significantly higher than the neutral scale value of 4. HUC resulted in higher average scores on the adapted exercise assessment scale (TAD4-5) compared to the video- and paper-based instructions.

The qualitative feedback suggested that the three instruction methods provided different levels of richness of information. Eight patients described the paper exercises as boring and not enjoyable to follow. Even though patients found the paper instructions helpful to recall the exercise, they often had trouble interpreting the instructions correctly. Three patients found the video instructions authentic and found the exercise easy to understand. However, patients mentioned that they felt insecure about the correctness of exercise execution. Patients also found the exercises easier to understand while using HUC and appreciated the guidance, the reminder app, and the real-time feedback to understand when mistakes were made. Eight patients also perceived HUC as fun and enjoyable. Six patients particularly enjoyed performing the exercise together with a life-sized CA. One patient, however, found that the CA did not engage emotionally. A disadvantage of HUC perceived by 9 patients—thereby a frequently perceived disadvantage—was the bulkiness of the AR hardware used in this study.

**Figure 9:**
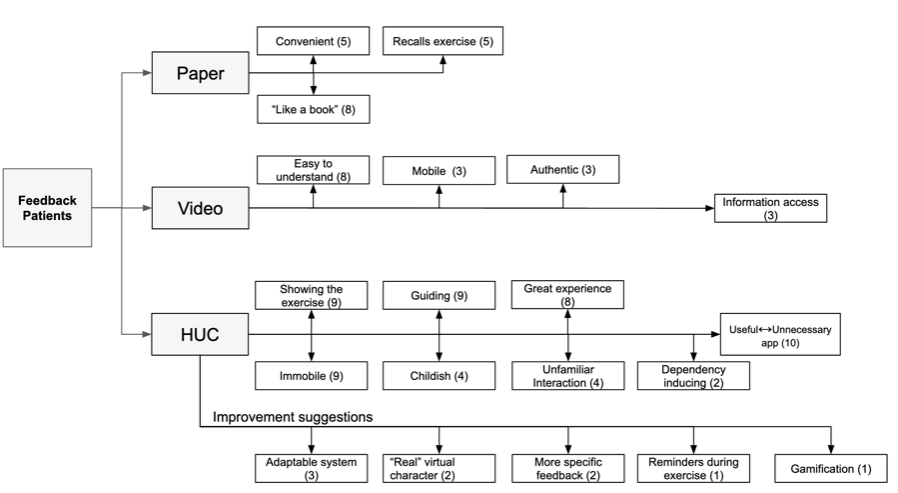
Thematic map of the patients' qualitative feedback of study 3. Note: the number in brackets indicates the frequency a topic was mentioned. HUC: hybrid ubiquitous coaching.

The patients suggested several improvements (Figure 9). First, 3 patients wanted to personalize the CA (eg, adjust the speed and the movement parameters to account for varying abilities, individually decide the appearance of the coach). Second, 2 patients mentioned a preference for a real person and not an animated character (eg, *“looking a bit more human-like, not such a computer-figure”).* Third, 2 patients suggested adding more detailed real-time feedback and reminders and elements of gamification (eg, rewards for regularity, real-time reminders, and accuracy). Overall, HUC was positively perceived.

**Table 4:** HUC-based, video-based, and paper-based exercise instruction methods assessed by 15 physiotherapy patients and exercise assessment scale assessed by 2 physiotherapists. Note: _ represents the exercise instruction method.

Construct		(Exemplary) item	n	HUC^a^ M^b^ (SD)	*P* value^c^	Video M (SD)	*P* value^c^	Paper M (SD)	*P* value^c^
**Task load linked to exercise instruction method^d^**									
	Mental capacity	How much mental and perceptual activity was required with _?	1	2.66 (1.11)	.005	3.06 (1.43)	.04	4.00 (1.36)	.75
	Frustration	How frustrated did you feel during the execution with the _?	1	2.66 (1.44)	.005	2.60 (1.24)	.004	2.80 (1.69)	.02
**Perceived characteristics of the instruction method^e^**									
	Perceived enjoyment	_ was fun to use.	1	5.26 (1.90)	.06	5.26 (1.09)	.005	4.13 (1.06)	.91
	Perceived ease of use	I could understand _ very easily.	1	6.20 (1.01)	.001	5.80 (1.08)	.002	5.53 (1.40)	.005
	Perceived usefulness	_ helped me to do the exercises correctly.	3	5.59 (1.63)	<.001	5.07 (1.52)	<.001	4.76 (1.42)	.002
**Patient–conversational agent working alliance^e^**									
	Goal agreement	Alex and I agree on what is important for me to work on.	2	4.89 (1.96)	.06	N/A^f^	N/A	N/A	N/A
	Task agreement	The way Alex and I are working with my problem is correct.	1	5.14 (1.51)	.02	N/A	N/A	N/A	N/A
	Attachment bond	Alex and I respect each other.	3	5.57 (1.45)	<.001	N/A	N/A	N/A	N/A
	Total		6	5.27 (1.65)	<.001	N/A	N/A	N/A	N/A
Intention to continuously use^e^		How much would you like to continuously use the [instruction method]?	1	5.07 (1.94)	.10	5.14 (1.29)	.01	5.14 (0.86)	.002
Exercise assessment scale^g^		Correct body part moving in correct plane	4	4.23 (0.75)	<.001	4.17 (1.06)	<.001	3.63 (0.90)	<.001

^a^HUC: hybrid ubiquitous coaching.

^b^M: mean.

^c^Wilcoxon signed rank test with a test value of 4 was used for all constructs but the exercise assessment scale, where a test value of 3 was used.

^d^7-point Likert scales ranging from very low (1) to very high (7).

^e^7-point Likert scales ranging from strongly disagree (1) to strongly agree (7).

^f^N/A: not applicable.

^g^Exercise assessment scale items resulted in a score from completely incorrect (0) to completely correct (6).

### Study 4: HUC Assessed in a 4-Week Field Study

#### Frequency and Engagement

**Figure 10:**
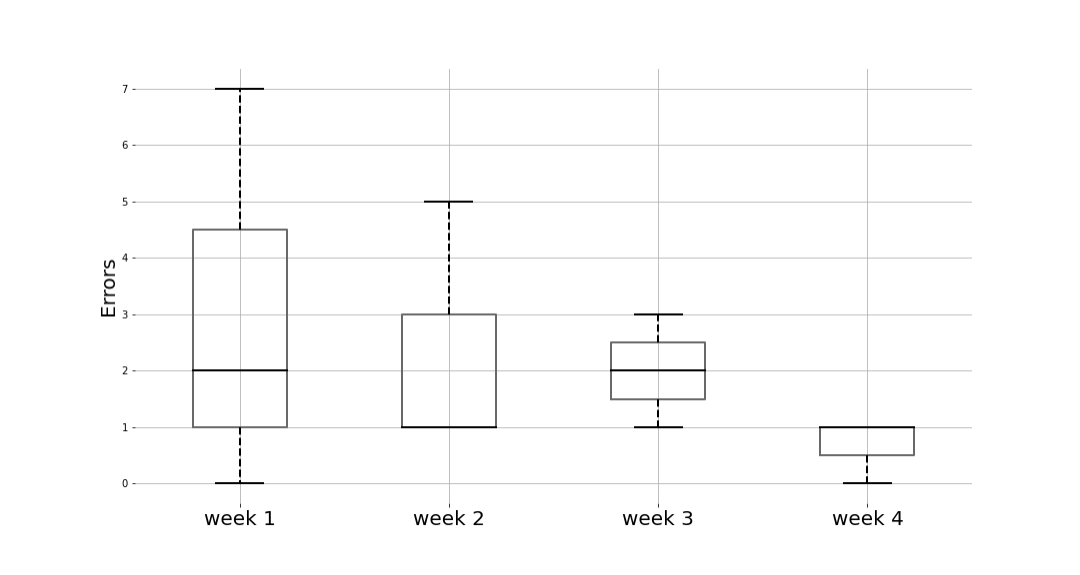
Box plot of the exercise execution errors during the 4 weeks. The number of errors was aggregated for each week.

Based on the behavioral data recorded by the HUC, a total of 3 exercise sessions per week were performed in 3 out of 4 weeks. This resulted in TAD rates of 92% in frequency, sets, and repetitions (Table 5). During the last week, the patient was on vacation and did not take the hardware with them. Therefore, only 2 sessions were performed. Not going low enough with the body during the squat exercise was the most common mistake. The average number of mistakes during the 4 weeks is depicted in Figure 10. The number of mistakes fell over the 4 weeks, indicating that the HUC’s real-time feedback was processed by the patient and, at least to some degree, put into action. In total, 17 standardized text messages were sent to remind the patient to perform the exercises, to inform the patient about their progress, and to provide motivational information about the importance of performing the exercises. The patient acknowledged these messages with 8 answers (Multimedia Appendix 12).

**Table 5:** Behavioral data from the 4-week HUC practice (N=1).

Construct	Value
Session completion rate, n (%)	11 (92)
Repetition rate, n (%)	330 (92)
Sets, n (%)	33 (92)
Errors (1st three sets), n (%)	8 (89)
Errors (last three sets), n (%)	4 (44)
HUC^a^ messages, mean (sum)	4.25 per week (17 in total)
Patient messages, mean (sum)	2 per week (8 in total)
Duration (s), mean (sum)	191 per session (2098 in total)

^a^HUC: hybrid ubiquitous coaching.

#### Debriefing Interview

Overall, the patient described the experience with the HUC as very positive. The exercises guided by the AR-based CA were enjoyable and motivating. The patient liked the feeling of not being alone while performing the exercises and remarked that they preferred the HUC to a human personal trainer, as it was perceived as being “more relaxing.” Additionally, the patient pointed out usually having difficulties adhering to home exercises and consequently appreciating the variations of the WhatsApp-based motivational messages from the smartphone-based CA and feedback on the execution accuracy by the AR-based CA, both of which helped the patient with adherence. The patient expressed an interest in having a more dynamic intervention program that includes longer sessions and variations in the exercises. Finally, the patient wanted to continue working with the smartphone-based and AR-based CA beyond the period of this n-of-1 trial.

#### Follow-up Interviews With Physiotherapists

Reflecting on the results of the study, all physiotherapists confirmed that HUC has the potential to improve adherence to home-based physiotherapy exercises. Referring to the progression of the exercise technique, the real-time feedback about the temporal and spatial accuracy was seen as a clear advantage. Two physiotherapists also liked the fact that the system ensured adherence to the prescribed number of sets and repetitions and described it as having a positive influence on patient motivation. As a treatment provider, the detailed monitoring information regarding the exercise technique was considered to be highly valuable. All physiotherapists highlighted that the therapy could be optimized and a patient’s problems could be addressed more individually during on-site therapy sessions. This could potentially also render the face-to-face encounters more efficient. In a future version of HUC, patients should be able to give feedback on their level of pain or well-being. Particularly, information about a patient’s pain level could enable more personalized digital coaching. Lastly, according to 2 therapists, it should be possible to calibrate the exercises more individually in terms of exercise technique by adding different variations of one exercise.

## Discussion

### Principal Findings

The goal of this paper was to address the problem of nonadherence to home exercises by proposing HUC, a human- and CA-supported coaching approach that employs smartphone and AR technology. This paper is, to the best of our knowledge, the first to investigate the potential of HUC with patients and health care experts in two lab studies, interview studies, and a longitudinal n-of-1 trial in the field. HUC required collaborative and interdisciplinary effort from various stakeholders, including health care experts, patients, and experts in behavioral medicine, game design, AR, and human computer interaction. A strength of this work and HUC is, therefore, that its pragmatic and diverse investigation helps to broadcast the results to real-world scenarios and inform practitioners about effective digital designs for addressing the problem of treatment adherence. Moreover, the implemented home exercise (ie, the squat) is not only relevant to physiotherapy patients but also an important component of other treatments, for example, of high-intensity interval trainings for patients who are overweight or patients with cardiovascular disease. In the following section, the results will be discussed along the research questions and design challenges. Limitations and future work will also be outlined.

### Research Question 1a: How is HUC Perceived by Patients?

Overall, patients stated that they enjoyed executing the exercise together with HUC, that they intended to use it, and that they would recommend the final HUC version to other patients. Compared to paper- and video-based instructions, HUC reached higher overall acceptance rates, required less mental capacity, was perceived as being easier to follow, and resulted in higher exercise accuracy ratings. However, significant differences were only found for the items *mental capacity*, *perceived enjoyment*, and *perceived usefulness* between HUC and the paper-based instructions. This is still promising, however, for the following reasons. First, HUC was rated at least as good as video instructions, which is encouraging, since video instructions are associated with reduced symptoms in physiotherapy [103]. Second, perceived enjoyment was significantly higher than with paper-based instructions, a promising result, since enjoyment is related to engagement in digital health interventions [104], something that is often not taken into consideration [105] and is important for long-term adherence. This was confirmed in the 4-week field study. In line with previous findings in the field of CAs [47,49,57,59], patients reported that they were able to build a working alliance with the CA. This was especially the case for attachment bonds, which include factors such as mutual trust, acceptance, and empathy [49,106]. Lastly, in the field study, the patient indicated that they were more motivated to do the exercise when using HUC. The positive perception of HUC and the feeling of collaboration between the patient and the smartphone- and AR-based CA implies an established working alliance.

In summary and to answer RQ1a, we conclude that patients accepted and enjoyed HUC. Patients were also able to build a working alliance with the CA.

### Research Question 1b: How is HUC Perceived by Health Care Experts?

HUC was also evaluated by physiotherapists to gather their feedback. Collecting behavioral data about various treatment adherence dimensions (eg, the session completion rate, the sets rate, and the exercise repetition rate) was seen as a clear advantage of HUC. Physiotherapists also considered the real-time feedback to be an additional motivator due to its personal, interactive, and gamified approach, which reflects an improvement relative to the status quo. However, physiotherapists were initially (study 2) more skeptical about whether HUC could be conducive to building a strong working alliance between patients and physiotherapists. The importance of personal interaction between the physiotherapist and the patient was one reason for the negative assessment of attachment bond. Physiotherapists expressed that mutual trust, empathy, and, consequently, mutual goals and tasks were uniquely established during face-to-face encounters. Nevertheless, both the quantitative and qualitative patient feedback (study 3) underline the potential that a strong working alliance can be built with a CA. This result is consistent with prior work about working alliances and CAs [68]. There is a possible explanation for the deviation between the working alliance ratings of physiotherapists (study 2) and patients (study 3): While patients assessed the working alliance with the CA, physiotherapists assessed it in comparison to the bond that develops during physical interaction between patient and therapist. However, HUC foresees the integration of human and CA coaching into both in-person and remote physiotherapy sessions (Figure 1). As HUC does not replace but rather complements the bond between the physiotherapist and patient, it is more equitable to compare HUC with traditional coaching methods. Thus, compared to conventional methods, HUC is shown to have the ability to increase the working alliance.

In the follow-up interviews about the field study, all physiotherapists agreed that home exercises in physiotherapy could be optimized with HUC. They were convinced that various treatment adherence dimensions could be addressed more efficiently with HUC.

In summary and to answer RQ1b, we conclude that HUC represents a clear relative advantage to current methods, as indicated by physiotherapists, in particular, with respect to its motivational features and real-time feedback.

### Research Question 2: Does HUC Lead to Treatment adherence to Home Exercises?

Results from study 3 and the 4-week n-of-1 trial were promising with respect to RQ2. Qualitative feedback from study 3 indicated that HUC addressed adherence challenges better than the paper- and video-based instruction methods. In particular, patients found the reminders (TAD1-2) useful, appreciated receiving guidance (TAD3) and real-time feedback on the spatial and temporal accuracy of the exercise execution (TAD4-5), and felt more comfortable about the exercise. Moreover, the results of the 4-week trial revealed a clear decrease in exercise execution errors (TAD4-5) and a high adherence rate of 92% in TAD1-3. Lastly, the patient reported being motivated and committed to doing the exercise doing the 4 weeks due to the variations in the messages and the feedback on exercise accuracy. Motivation and enjoyment are targeted by HUC and, according to prior work, associated with significantly increased rates of adherence [14,29,30].

In summary and to answer RQ2, we conclude that HUC did address the session completion rate (TAD1), set completion rate (TAD2), exercise repetition rate (TAD3), and temporal and spatial exercise accuracy (TAD5). Although this study does not prove beyond a doubt that the use of HUC led to better adherence, the rate was far higher than has typically been found in previous studies [18,20] and was also higher than the participant's previous adherence to home physiotherapy exercise based on their self-report of their own practice and difficulties.

### Design Challenges

A first challenge during the design of HUC was the development of the CA’s personality in such a way that the patient perceives the CA coherently as the physiotherapist’s digital assistant via text messages on the smartphone and voice messages in the AR space. The first lab study and the NPS revealed that HUC, and the design in particular, still needed improvement. Accordingly, the first version of HUC was modified based on this feedback. In the second lab study, patients reported correspondently higher levels of satisfaction with HUC and acceptance of the design of the CA. Further, another challenge was the design of the AR interactions, which had to take into account the limited exercise space at patients’ homes, the limited field of view of the AR glasses, and anthropometric characteristics of patients. Any additional controller hardware also had to be omitted due to the requirement that physiotherapy exercises be hands-free. This challenge was partly met. Patients ranked HUC as their first-choice coaching method but still commented on the bulkiness of the hardware. Another major challenge was the design of variations in the smartphone-based and AR-based CA conversations to improve long-term adherence to physiotherapy in the last study. Variations in the design were implemented to make the exercises more diversified and to thereby increase adherence. The positive result for treatment adherence indicated that the last major design challenge was met.

### Limitations and Future Work

The current work has the following limitations. First and foremost, in the current HUC version, only one home exercise was implemented, and thus the findings, especially those related to the AR-based CA, cannot be generalized to a large extent. Implementations of HUC should be further developed to reach a level of implementation foreseen in its approach, as outlined in Figure 1. Future work is therefore required to develop an intuitive CA that supports health care experts through the customization of home exercise sessions, for example, via suggestions given by prior patients [107,108]. Additionally, the problem with the deviations in the exercises could also be solved with machine learning. In doing so, exercise suggestions could be learned, for example, from a basket analysis that considers past exercise programs, patient-related variables (eg, diagnosis and preferences) and past treatment outcomes (eg, reduction in pain) [109,110].

Second, objective adherence rates were based on lab studies and a 4-week study. Regular physical therapy treatment lasts approximately 9 weeks, while long-term treatment can last more than 9 months (36 sessions) [111]. Thus, HUC needs to be assessed not only by a representative sample of patients but also for a representative treatment duration that is common not only in physiotherapy but also in other treatments that require intensive home exercises (eg, treatments for patients with obesity or cardiovascular disease).

Third, the current AR-based CA had a fixed coaching approach. A growing body of research reveals that certain personalities and therapeutic techniques (eg, interpersonal, person-centered, behavioral) work better in specific contexts [112,113]. The suitability of the coaching approach can also depend on individuals’ personality traits [114]. By tailoring the coaching technique, the CA could be perceived as more empathetic, potentially making the coaching experience more enjoyable and thereby increasing both adherence and the working alliance and with them, treatment outcomes [112]. Further, we did not investigate whether the prescribed exercises actually had an effect on health outcomes. An adaptive algorithm that increases the difficulty, repetition, and intensity of the exercises could be implemented to ensure that the patient always performs slightly above the comfort zone. This might have an effect not only on health outcomes, but also on adherence. Lastly, the AR-based part of HUC was not yet perceived to be mobile enough, and this was a frequently expressed reason why patients were doubtful about using HUC. Nevertheless, this study was first and foremost about testing real-time feedback and a hands-free interaction paradigm with a “human-sized” CA. The AR hardware is currently changing and shrinking significantly [115], and future work will therefore rely on significantly smaller AR and will be able to test HUC on sufficiently more wearable hardware.

### Conclusions

This work provides evidence of the relevance and utility of HUC that aims to increase adherence to home exercises. It therefore contributes to the field of digital health by outlining how CAs hosted by mobile and wearable technologies can extend the reach and effectiveness of health care experts into the everyday lives of patients. HUC may be promising not only in the context of physiotherapy, as exemplarily elaborated in this work, but also for various other conditions, such as obesity or cardiovascular disease, as they also require intensive and longitudinal behavioral support in the everyday lives of patients.

## Appendix

Multimedia Appendix 1 Current State Commercial and Research Applications.

Multimedia Appendix 2 Storybook of the hybrid ubiquitous coaching approach.

Multimedia Appendix 3 First-person view and interactions with the augmented reality-based conversational agent version 1 of study 1.

Multimedia Appendix 4 Technical details of the augmented reality-based conversational agents versions 1 and 2.

Multimedia Appendix 5 First-person view and interactions with the augmented reality-based conversational agent version 2.

Multimedia Appendix 6 Sketches of the hybrid ubiquitous coaching approach.

Multimedia Appendix 7 Questions of the semistructured interview of study 2.

Multimedia Appendix 8 Video-based instructions of study 3.

Multimedia Appendix 9 Quantitative questionnaire items and depersonalized raw data of all 4 studies.

Multimedia Appendix 10 Questions of the semistructured interview of study 3.

Multimedia Appendix 11 Data analysis script written in Python for all 4 studies.

Multimedia Appendix 12 Examples of the smartphone-based conversational turns of study 4.

## Abbreviations

AR: augmented reality
BCT: behavioral change technique
CA: conversational agent
HUC: hybrid ubiquitous coaching
MSD: musculoskeletal disorder
NPS: Net Promoter Score
QR: quick response
RQ: research question
TAD: treatment adherence dimension
