# Supplementary material for: Hybrid Ubiquitous Coaching With a Novel Combination of Mobile and Holographic Conversational Agents Targeting Adherence to Home Exercises: Four Design and Evaluation Studies
Source: J Med Internet Res. 2021 Feb 22;23(2):e23612. doi: 10.2196/23612 (PMC7939948; doi:10.2196/23612)
Supplement: Multimedia Appendix 1 [file jmir_v23i2e23612_app1.docx]

# Multimedia Appendix 1 - Current State of Commercial and Research Applications

We reviewed recent commercial and research applications in physiotherapy to better understand the current state of the art with respect to treatment adherence. Pitchbook, Crunchbase Pro, Google search, Google Play, and Apple’s App Store were used to identify relevant remote patient monitoring tools and telehealth services. PubMed, Science Direct, and Google Scholar were used to identify relevant peer-reviewed scientific articles. Search terms were related to the type of therapy (i.e., physiotherapy, physical therapy, and rehabilitation), treatment location (i.e., home, home-based, remote), and the mode of delivery (i.e., mixed reality, MR, virtual reality, VR, augmented reality, AR, digital, web, internet, telecare, app, mobile, mobile phone, smartphone, computer, tool). We further defined the following eligibility criteria. First, commercial applications had to be on the market and offered to either physiotherapists or directly to patients. Second, scientific articles describing relevant research applications were included if they had been published from 2015 onwards. Third, applications for sole prevention purposes of MSDs or general fitness and sport interventions were excluded since they do not have a medical pretense as required in physiotherapy. Applications for sole in-practice use under the surveillance of a physiotherapist were excluded as they do not aim to provide support between face-to-face encounters. Applications addressing other health-related disorders, e.g., anxiety, were excluded. The final search was conducted in July 2020. All commercial applications and scientific work were screened for eligibility and assessed by two co-authors. The following assessment categories were used: CA (e.g., either text-based or video-based)^[[1]](#footnote-1)^, mode of delivery (e.g., educational instructions via paper, video, or VR), real-time feedback, everyday coaching (e.g., receiving reminders, psychoeducational material, and motivational messages), data exchange with physiotherapists to increase the working alliance (e.g., what type of information and whether data is transferred automatically or manually), existence of a physiotherapist-patient communication channel, TADs targeted, and impact on primary health outcomes (e.g., significant reduction in back pain).

Table 1. Assessment of commercial and research applications for home exercises

| Sources | CA^a^ | EI^b^ | RTF^c^ | EC^d^ | Data Exchange | | CC^e^ | TAD^f^ | IHO^g^ |
| --- | --- | --- | --- | --- | --- | --- | --- | --- | --- |
|  |  |  |  |  |  | |  |  |  |
| **Commercial applications** | |  |  |  |  | |  |  |  |
| Mirareb | Yes | Video | TAD2-4 | No | TAD1, Pain, Rating | A^h^ | Video | TAD1-5 | - |
| Reflex | No | Video | TAD2-4 | RM | Pain, TAD1-5, ROM | A | Video | TAD1-5 | - |
| CASPAR[1] | No | Video | No | RM^i^, PM^j^, MM^k^ | ANS^l^, Video | M^m^ | Text | - | - |
| Sophy app[2] | No | Video | No | RM | TAD1, Pain | M | No | - | - |
| Physitrack[3,4] | No | Video | No | RM, PM | TAD1-2, Pain | M | TextVideo | TAD1-3 | - |
| ViViRA[5] | Text | Video | No | RM, PM | - | - | No | - | - |
| Healure[6] | No | Video | No | RM | ANS, Pain, Mood | M | No | - | - |
| Pt Pal Pro[7] | No | Video | No | RM, PM | TAD1, Pain, Dif.^n^ | M | Text | - | - |
| MedBridge[8] | No | Video | No | RM, PM | TAD1-2, Pain, Dif. | M | Text | - | - |
| Physiotools[9] | No | Video | No | RM, PM | - | - | No | - | - |
| Salaso[10] | No | Video | No | RM, PM | TAD1-2, Effort, Pain | M | Text | - | - |
| BlueJay[11] | No | Video | No | RM, PM | TAD1-3, Pain, Video | M | Text | - | - |
| Physioplux[12] | No | Video | TAD2-4 | No | TAD1-4 | A | No | - | - |
| Valedo[13,14] | No | Video | TAD2-5 | No | TAD1-5 | A | No | - | LMC↑^o^ |
| SWORD[15,16] | No | Video | TAD2-5 | PM, MM | TAD1-5, Pain, Video | A | Text | - | TUG↑^p^ ROM↑^q^ KOOS^r^ |
| Kaia[17,18] | No | Video | TAD3-5 | RM, PM, MM | - | - | No | TAD1 | BP^s^ GCPS^t^ PMW^u^ |
| XR Health[19] | No | VR^v^ | TAD2-5 | No | TAD1-5 | A | No | - | - |
| Verapy[20] | No | VR | - | No | - | - | - | - | - |
| VeraHome[21,22] | Yes | Video | TAD2-5 | No | TAD1-5 | A | No | TAD1-3 | KOOS |
| **Research applications** | |  |  |  |  |  |  |  |  |
| Clark et al. [23] | No | Video | No | RM, PM | TAD1-3 | M | No | TAD1-5 | SE^w^  Pain |
| Lambert et al. [24] | No | Video | No | RM, MM | TAD1 | M | Text/ Call | SRA↑ | PSFS↑^x^ SHODAS^y^ |
| Lo et al. [25] | No | Video | No | RM, PM | - | - | No | - | - |
| Tang et al. [26] | No | ANIM^z^ | TAD5 | No | TAD5 | A | No | - | - |
| Timmers et al. [27] | No | Video | No | RM, PM | - | - | No | TAD1 | KP^zz^, KOOS |
| Stütz et al. [28] | No | Video | No | Calendar | TAD1-3, Mobility | A | No | - | - |

^a^Conversational agent, ^b^Educational instructions, ^c^Real-time feedback, ^d^Everyday coaching, ^e^Coaching channel, ^f^Treatment adherence dimension (see Table 1), ^g^Inferior health outcomes, ^h^Automatic data exchange, ^i^Reminder, ^j^Psychoeducational material, ^k^Motivational messages, ^l^Adherence dimension not specified, ^m^Manual data exchange, ^n^Difficulty, ^o^Lumbopelvic movement control, ^p^Mobility test, ^q^Range of motion, ^r^Knee Injury & Osteoarthritis Outcome Score, ^s^Back pain, ^t^Graded Chronic Pain Scale, ^u^Physical & Mental Scale, ^v^Virtual reality, ^w^Shoulder Function, ^x^Patient-specific-function-scale, ^y^World Health Organization Disability Assessment Schedule, ^z^Animation, ^zz^Knee pain. Note: only random controlled trials indicating significant improvements in at least one TAD or health outcome are listed.

A total of 19 commercial applications (76%) and six research applications (24%) were identified (See Table 1). Three used a CA (12%), and videos were the most frequently used educational instruction tool (22, 88 %). Nine applications provided real-time feedback on various TADs, including exercise repetition rate, set completion rate, and spatial and temporal accuracy (36%). Moreover, the majority of applications provided everyday coaching (18, 72%), of which reminders were most frequently used (16, 64%), and data exchange (e.g., TADs, pain level, exercise rating) between patients and physiotherapists (19, 76%). However, the data exchange was only automated in nine applications (36%) and only ten applications provided a coaching channel to allow communication between the physiotherapist and patient (40%). Seven sources included a study on the application’s ability to address TADs and health outcomes (28%).

In summary, only three applications included a CA, of which one increased adherence in a RCT [6] through reminders. There exist various applications that aim to increase several TADs to home exercises. However, currently, no identified application provided a holistic approach like the one we have proposed with Hybrid Ubiquitous Coaching (HUC).

## References

1. Healure Technology. Healure: Physiotherapy Exercise Plans. Published online 2017. http://www.healure.com/

2. Cassell J. More Than Just Another Pretty Face : Embodied Conversational Interface Agents. *Commun ACM*. 2000;43:70-78. doi:10.1145/332051.332075

3. Laranjo L, Dunn AG, Tong HL, et al. Conversational agents in healthcare: A systematic review. *J Am Med Informatics Assoc*. 2018;25(9):1248-1258. doi:10.1093/jamia/ocy072

4. de Cock C, Milne-Ives M, van Velthoven MH, Alturkistani A, Lam C, Meinert E. Effectiveness of Conversational Agents (Virtual Assistants) in Health Care: Protocol for a Systematic Review. *JMIR Res Protoc*. Published online 2020. doi:10.2196/16934

5. Fitzpatrick KK, Darcy A, Vierhile M. Delivering Cognitive Behavior Therapy to Young Adults With Symptoms of Depression and Anxiety Using a Fully Automated Conversational Agent (Woebot): A Randomized Controlled Trial. *JMIR Ment Heal*. 2017;4(2):e19. doi:10.2196/mental.7785

6. Kowatsch T, Nißen M, Shih C-HI, et al. Text-based Healthcare Chatbots Supporting Patient and Health Professional Teams : Preliminary Results of a Randomized Controlled Trial on Childhood Obesity. *Persuas Embodied Agents Behav Chang Work*. 2017;1(Iva 2017):1-10. www.mobile-coach.eu

7. Kramer J-N, Künzler F, Mishra V, et al. Which Components of a Smartphone Walking App Help Users to Reach Personalized Step Goals? Results From an Optimization Trial. *Ann Behav Med*. Published online March 17, 2020. doi:10.1093/abm/kaaa002

8. Hauser-Ulrich S, Künzli H, Meier-Peterhans D, Kowatsch T. A smartphone-based health care chatbot to promote self-management of chronic pain (SELMA): pilot randomized controlled trial. *JMIR mHealth uHealth*. 2020;8(4):e15806. doi:10.2196/preprints.15806

9. Prvu Bettger J., Green CL, Holmes, DN et al. Effects of Virtual Exercise Rehabilitation In-Home Therapy Compared with Traditional Care After Total Knee Arthroplasty. *The Journal of Bone and Joint Surgery*. 2020 102(2):101-109. doi:10.2106/JBJS.19.00695

10. Vivira Health Lab GmbH. Vivira - Physiotherapy Exercises at Home. Published online 2016. https://www.vivira.com/

11. Ma T, Chattopadhyay D, Sharifi H. Virtual humans in health-related interventions: A meta-analysis. In: *Conference on Human Factors in Computing Systems*; 2019:1-6. doi:10.1145/3290607.3312853

12. Babylon Health. Babylon. Published online 2016. https://www.babylonhealth.com/

13. Bickmore TW, Pfeifer LM, Byron D, et al. Usability of Conversational Agents by Patients with Inadequate Health Literacy: Evidence from Two Clinical Trials. *J Health Commun*. 2010;15(S2):197-210. doi:10.1080/10810730.2010.499991

14. Verapy Health. Verapy. Published online 2016. https://verapytherapy.com/

15. GOREHA GmbH. XR Health. Published online 2018. https://www.xr.health/

16. Nass C, Steuer J, Tauber ER. Computer are social actors. In: *Conference on Human Factors in Computing Systems - Proceedings*. 1994:72-78. doi:10.1145/259963.260288

17. Bickmore T, Gruber A, Picard R. Establishing the computer-patient working alliance in automated health behavior change interventions. *Patient Educ Couns*. 2005;59(1):21-30. doi:10.1016/j.pec.2004.09.008

18. Shamekhi A, Bickmore T, Lestoquoy A, Gardiner P. Augmenting group medical visits with conversational agents for stress management behavior change. In: *Persuasive Technology: Development and Implementation of Personalized Technologies to Change Attitudes and Behaviors: : 12th International Conference, PERSUASIVE 2017*. Vol 10171. Springer; 2017:55-67. doi:10.1007/978-3-319-55134-0_5

19. Flückiger C, Del Re AC, Wampold B, Horvath AO. The Alliance in Adult Psychotherapy: A Meta-Analytic Synthesis. 2018;55(4):316-340. doi:10.1037/pst0000172

20. Cassell J, Sullivan J, Prevost S. *Embodied Conversational Agents Edited By*. MIT Press; 2000.

21. Campbell AG, Stafford JW, Holz T, O’Hare GMP. Why, when and how to use augmented reality agents (AuRAs). *Virtual Real*. 2014;18(2):139-159. doi:10.1007/s10055-013-0239-4

22. Milgram P, Takemura H, Utsumi A, Kishino F. Augmented reality: a class of displays on the reality-virtuality continuum. 1995;2351:282-292. doi:10.1117/12.197321

23. Bassett S. Bridging the intention-behaviour gap with behaviour change strategies for physiotherapy rehabilitation non-adherence. *New Zeal J Physiother*. 2015;43(3):105-111. doi:10.15619/nzjp/43.3.05

24. Bandura A. Self-efficacy mechanism in human agency. *Am Psychol*. 1982;37(2):122.

25. Schwarzer R. Modeling health behavior change: How to predict and modify the adoption and maintenance of health behaviors. *Appl Psychol*. 2008;57(1):1-29. doi:10.1111/j.1464-0597.2007.00325.x

26. Andersson G, Carlbring P, Berger T, Almlöv J, Cuijpers P. What Makes Internet Therapy Work? *Cogn Behav Ther*. 2009;38(sup1):55-60. doi:10.1080/16506070902916400

27. Spek V, Cuijpers P, NykIíček I, Riper H, Keyzer J, Pop V. Internet-based cognitive behaviour therapy for symptoms of depression and anxiety: A meta-analysis. *Psychol Med*. 2007;37(3):319-328. doi:10.1017/S0033291706008944

28. Zachariae R, Lyby MS, Ritterband LM, O’Toole MS. Efficacy of internet-delivered cognitive-behavioral therapy for insomnia - A systematic review and meta-analysis of randomized controlled trials. *Sleep Med Rev*. Published online 2016. doi:10.1016/j.smrv.2015.10.004

1. A conversational agent (CA) is a computer program that imitates communication with a human being [2]. Due to their scalability, CAs have become popular in various healthcare applications [3,4] targeting depression and anxiety [5], childhood obesity [6], physical activity [7], chronic pain management [8], and physiotherapy [9,10]. A first meta-analysis has also shown their effectiveness both in clinical and non-clinical randomized controlled trials [11]. CAs deliver their interventions through various channels, such as websites [9,12], smartphones [6,7], tablets [13], virtual reality [14], or augmented reality [15]. Moreover, CAs are perceived as social actors[16] and, thus, are able to increase the working alliance with patients [17,18]. A working alliance between the patient and the digital therapist is an essential part of treatment adherence [6,19], and helps improve health outcomes [17]. With these characteristics, CAs can deliver relevant psychoeducational material about physiotherapy in the form of video clips or text messages (e.g., about the benefits of adherence behavior) and exercise reminders via smartphones into the everyday lives of patients. Moreover, virtual or holographic embodied CAs [20] have the potential to increase the spatial and temporal accuracy of exercises through real-time instruction, monitoring exercise behavior and, in turn, provision of real-time feedback about that behavior [14,15,21,22]. Real-time feedback can also increase self-efficacy [23], an important belief in one’s ability to perform a health-promoting behavior [24,25]. Finally, there is increasing evidence that a combination of human health coaches and digital (CA-based) interventions can increase treatment adherence [26–28]. [↑](#footnote-ref-1)
