# Supplementary material for: Hybrid Ubiquitous Coaching With a Novel Combination of Mobile and Holographic Conversational Agents Targeting Adherence to Home Exercises: Four Design and Evaluation Studies
Source: J Med Internet Res. 2021 Feb 22;23(2):e23612. doi: 10.2196/23612 (PMC7939948; doi:10.2196/23612)
Supplement: Multimedia Appendix 2 [file jmir_v23i2e23612_app2.docx]

# Multimedia Appendix 2 - Storybook of the hybrid ubiquitous coaching approach

**Alex - Your Physiocoach**

Version 3.1, August 31, 2018

**The Story**

1. I am Frank 🧓. I live close to Zurich, Switzerland 🇨🇭, like 20 minutes by train or so. I am a desktop worker 🖥 and I have serious back pain since a couple of weeks 😫. Yesterday, I lifted my 7-year-old daughter 👨‍👧 and 😫 ahhhhhh 😫 in a way that I almost can’t move my body anymore. My back pain is beyond all bearing now 😫😫😫.
2. I went to my GP due to the recommendation of my health insurers I got 9 hours of physiotherapy prescribed. I didn’t like the idea that I had to go and travel several times to that physiotherapy, which was in Zurich, because I really love to be every second with my family when I am not in my office. Nevertheless, I had to go because I had no choice due to my severe back pain.
3. I then had my first hour with Lucas in Zurich, long-standing expert in physiotherapy 💪. I have to admit, already after a couple of minutes with him, I really liked his approach helping me with my back pain and I felt the progress from hour to hour. He is a world champion in motivating me doing the exercises correctly with all repetitions that are required when I was with him in Zurich.
4. However, at home, I sometimes forgot to do these exercises and I was often not sure whether I did these exercises correctly because Lucas was not present, and I am not able to afford a personal trainer helping me out at home 😉.
5. After a couple of on-site physio lessons, I asked Lucas whether there would be an easy way to get reminders about and support with the important physio exercises at home 🧐. Lucas smiled at me 🙂 and told me:

   “Yes, I think we have a nice solution for you Frank 👍. I have already planned to tell you about my digital coach ALEX that helps me to **deliver my personalized physio exercises at the right moment to you at home**. I just wanted to wait for some lessons with you to better understand your individual characteristics and thus being able to tailor the support of ALEX perfectly to your personal needs.”

   “Wow, what do you mean with a digital coach ALEX?” I was asking.

   “Well, ALEX is our digital physio coach, aka a conversational agent or chatbot. I can give you access to it so that ALEX helps you with your exercises at home. BTW: You can choose among ALEXA or ALEX, just as you prefer 😁.” Lucas told me:

   I asked then: “Well, but how does it work? How would ALEX support me?”

   Lucas told me: “I will show you!” [Lucas walks to his laptop] “Look, I can easily customize the exercises that I recommend you to do at home via this exercise configurator, then I print a physio card for you with an individual QR code [Lucas shows an example of the physio card, in a traditional business card format]. This code links me as your instructor and master 😉 with your personal data such as **your name** or your **physiological constitution** such as height and weight and personal exercise preferences. With this information, **ALEX knows exactly how to talk to you and to tailor the exercises to you at home including an outcome measurement.**”

   I: “WOW 👍. Tell me more about it Lucas, I am very interested 🤗”

Lucas: “First, you will use your photo app on your smartphone to scan the QR code of your personal physio card. This link directs you to the ALEX app 📱 store. When you then download and start the app for the very first time, ALEX asks you to scan the QR code of your card once more and he or she will then welcome you and give you all the information that is needed at home. According to our therapy plan, ALEX will then **motivate** you **to do the exercises at the right moment in time** when you are able to receive and process our physio support. ALEX will also ask you from time to time about the **outcomes of our physio therapy like sleep quality, the degree of pain**, etc. And the really cool thing is that ALEX is then always with you and you **cannot forget the exercises anymore**. Or, put in other words, there is no excuse anymore to forget them 😉.

I: “Ha, I understand, really nice. But how would ALEX help me with doing the exercises correctly 🤔?

Lucas: “Well, ALEX does not only live in our ALEX app on your smartphone 📱 but can travel to another world in your home we call Mixed Realty 😀. You just need the AR device that my health insurer sends directly to you when I click this button here. You then need to just scan the barcode with your AR device to allow ALEX to welcome you in that mixed reality world, too.”

Lucas then showed me how ALEX would help me in doing the exercises with the AR device and I was completely flashed. Then Lucas told me: “Well, ALEX is a huge help for me, too. ALEX tells me when you do which exercises with how many repetitions including a quality score reflecting the **correctness of the exercise**. ALEX will also provide you real-time feedback so that you can increase that score on your own. Finally, **I am also way more efficient in our on-site lessons** in helping you **tailoring and optimizing the exercises** from time to time. And if you are doing it right and I am happy with your exercises, we **may drop one or two personal hours** as I get a compensation from the health insurance for you doing tele-coaching with you.”

I was really convinced about what Lucas told me and I wanted to test that approach instantly.

1. I got my first personal physio business card from ALEX and installed the ALEX app. Then, ALEX welcomed me and introduced himself as a digital coach of Lucas like in the following screenshot. This gave me a **feeling of trust** and **security**. It was like **one smooth kind of (clinical/physio/patient) pathway**. That felt really good. ALEX then chatted with me about the most important information to complement what Lucas has already told me in person. And then, it was at 5pm the next day, when ALEX told me via the ALEX app that my AR device will arrive in the next couple of minutes and that I will see ALEX soon for the very first time in the 3^rd^ dimension in the privacy of my home. Just thinking about that was really exciting and fun 😂. I then got the hardware and started it up as Lucas instructed me already back then in his office.


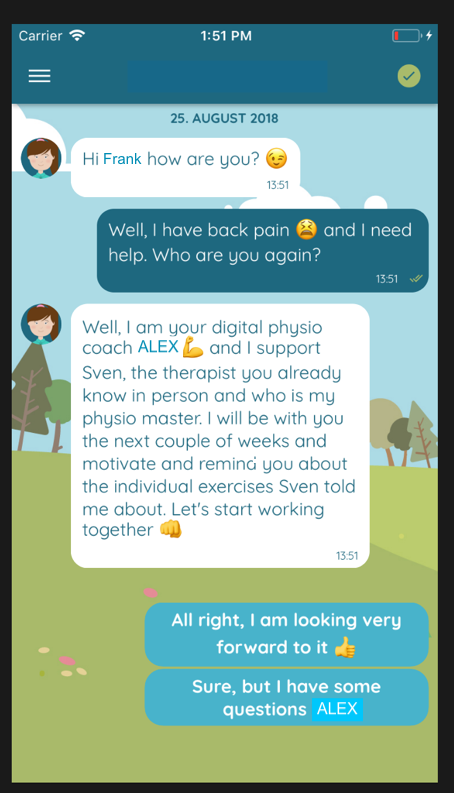


**Demo Instructions**

**Comment**

Frank wears the AR device without the control device. DO “It knocks 3 times like on an old wooden attic door, followed by 5s silence.” UNTIL “Frank looks down at the floor where the knocks should come from at a small 10cm by 10cm wooden door.” Then ALEX opens up the door, which makes a creaky sound, and starts to fly up and around the head of Frank showcasing the 3D sound effect and then keeps floating 30-50cm in front of Frank’s eyes. The distance between Frank’s eyes and ALEX’s eyes should be really close to when a real person would interact with someone to tap into existing communication patterns.

***About ALEX:*** ALEX has to have the very same character as on the smartphone. ALEX is small, like a tiny robot (e.g. PIXAR’s WALL-E or EVE-like) but definitely not visually perfect like EVE. There needs to be something broken and / or vulnerable to make the character loveable and authentic (like in FINDING NEMO the “small flapper” of Nemo >> we should be aware that we want to address 40+ individuals at the DEMO space, so maybe a more grown-up character might work quite well for them) as people do not like perfectness in that sense.

**ALEX**

Hi Frank, isn't it nice that we meet at eye level 👀?

By the way:

You can chat with me by touching
the answer options here [ALEX points towards a brick-like answer option] with your fingertip. Just try it out.

***Comment***

*I interact with ALEX by touching with my hand pre-defined answers like in the most successful game of all times or like the pre-defined answers in the Chat-App ALEX to be highly consistent and not to come up with too much new interaction paradigms. That is, we use of existing patterns: None / hardly any of the DEMO users has ever had an VR/AR/MR device. In 1-2 years, we may use more natural interaction paradigms. We must always be clear: For most people the DEMO in October will be a completely new experience. Also there will be a lot of noise and speech interaction / detection will probably not work there. By touching the answer blocks with the fingertip, visual (small animation that the message has been touch) and acoustic feedback (like in a chat app) should be given. ALEX should talk to me via written chat messages and with a pre-defined audio’s recorded (female and / or male voices as pre-defined during the onboarding dialog on the smartphone) due to the noise level and due to the fact that we try to drop any additional hardware that might be required (e.g. isolating headphones or the AR DEVICE Control device).*

**Frank**

Like so?

**ALEX**

Exactly, well done Frank!

How do you feel? 😉

**Frank**

Well, fine. Thanks!

So, what do we do now?

**ALEX**

Well, I support your physiotherapist Lucas [ALEX points with his finger toward a picture with a smiling and motivating Lucas saying in a speech bubble: “Hello”] and would like to help you now, that we get your back pain under control 👊

**Frank**

I'm skeptical, but Iet’s try it out ALEX 😉

**ALEX**

All right. Have a look at this video!

***Comment***

*There will be a "classic / "old-school" video clip showing a squat exercise with poor playback quality so that we have a nice delta that there is coming soon something different; for the suspense & surprise effect. ALEX stops the video after a couple of seconds and says:*

**ALEX**

Oh, I am so sorry 😂, I didn't want to show you that one. That's what my Grandma and Grandpa ALEX did with their clients in the 20th century. Give me just a moment please...

***Comment***

*The video screen dissolves into smoke / particles with an impressive audio-visual effect. And, accompanied by a whoosh sound effect, a 3D model called* ***Ken*** *appears, for example, as in a 3D print, layer by layer printed from the bottom to the top and/ or as the beaming sequence in Starship Enterprise. Again: we must use existing thought patterns, it must not be too new, otherwise the participants of the DEMO will not understand it. We are first and foremost interested to demo this to older people, too. So it has to be as always: easy to understand.*

**ALEX**

Yeah 😁. We are in the 21st century now.

I'll also start the animation of the exercise now.

Follow me and take a closer look at our physio model **Ken**.

***Comment***

*Ken begins to move and execute the following exercise in an endless loop. After a couple of seconds and with “Follow me and ...” ALEX slowly starts to flie circa 180 degress around the model while looking back to Frank. It will be evaluated whether Frank follows. If Frank follows, ALEX will provide positive feedback:
“You’re doing great! By doing so, you see the different perspectives of the exercise. This helps you to better understand how to do the exercise yourself in a second.”
If Frank does not follow, ALEX tries to motivate Frank to do so.
“Hey Frank, just follow me and I show you Ken and himself doing the exercise from another perspective.” This is for about 15 seconds.*

**ALEX**

What do you think, Frank?

**Frank**

Option 1: Wow, this is very impressive! 👍👍👍

Option 2: All right, interesting! 🤨

Option 3: Nice, but I am not that impressed! 😴

***Comment***

*The three answer options are used to evaluate this very first experience. After approx. 20 seconds, ALEX flies back in front of the participant's eyes and says:*

**ALEX**

[If answer option 1/2 was touched]

Hey Frank! So now it's your turn!

[If answer option 3 was touched]

Alright, so let's see if you can impress me
any better with your performance.😉

It's your turn now!
