# Supplementary material for: Hybrid Ubiquitous Coaching With a Novel Combination of Mobile and Holographic Conversational Agents Targeting Adherence to Home Exercises: Four Design and Evaluation Studies
Source: J Med Internet Res. 2021 Feb 22;23(2):e23612. doi: 10.2196/23612 (PMC7939948; doi:10.2196/23612)
Supplement: Multimedia Appendix 4 [file jmir_v23i2e23612_app4.docx]

# Multimedia Appendix 4: Technical details of the augmented reality-based conversational agents versions 1 and 2

**Technical details version 1:**

The AR-based HUC scenario was developed with the Unity game engine (2018.1.6f1-MLTP), using the Magic Leap SDK (v0.16.0). Blender (for 3D modeling), Audacity (for audio editing), and Photoshop (for texture editing) were additional tools used for the development. The AR headset Magic Leap One was chosen for the hardware. The headset contains a processing unit and a controller. Through integrated cameras, the device creates a virtual, three-dimensional representation of its surroundings, allowing for the placement of virtual objects into the user’s field of view. Thereby, the real-world spaces were integrated seamlessly. The device creates a world coordinate system that aligns with the direction of gravity. The output for each frame was the position and rotation of the headset and the controller within the coordinate system.

The algorithm for the automatic exercise recognition detects changes in the vertical axis (y-axis) of the headset, and velocity changes and compares them against threshold values: At the beginning of the exercise, the participant was asked to stand upright. The upright position of the headset was saved as the initial position. During the exercise, the position was tracked and updated continuously. The velocity was calculated for each frame by using the changes in position, and the time elapsed between two frames. The algorithm for the exercise recognition consisted of a sequence of three conditional statements:

1. Monitor if the headset exceeds a predefined threshold value (15 cm) down the vertical world-coordinate-axis (y-axis).
2. Check if the headset’s velocity on the y-axis approximates zero (expected velocity change at the lowest point) with a threshold value of 0.1.
3. Examine if the headset’s vertical position approaches its initial position with a threshold value of 10cm. The threshold values were defined and balanced through interviews and evaluated on two physiotherapists and seven test subjects.

**Technical details version 2:**

1. Insufficient depth of the exercise was indicated if a threshold value (Participants height multiplied with a fixed percentage value of 0.25) downwards was not exceeded.
2. Deviation of the speed of the exercise execution was detected if the time elapsed between two repetitions was either less than 1.5 seconds (too fast execution) or more than four seconds (too slow execution).
3. Deviation towards either side was detected if the distance on the horizontal world-plane between the initial and the current position exceeded predefined threshold values (left: 10cm, right: 10cm, back: 10cm, front: 30cm).
