# Supplementary material for: Hybrid Ubiquitous Coaching With a Novel Combination of Mobile and Holographic Conversational Agents Targeting Adherence to Home Exercises: Four Design and Evaluation Studies
Source: J Med Internet Res. 2021 Feb 22;23(2):e23612. doi: 10.2196/23612 (PMC7939948; doi:10.2196/23612)
Supplement: Multimedia Appendix 6 [file jmir_v23i2e23612_app6.pdf]

Physio in practice ~~therapist~~

① Exercise Program Selection Calibration

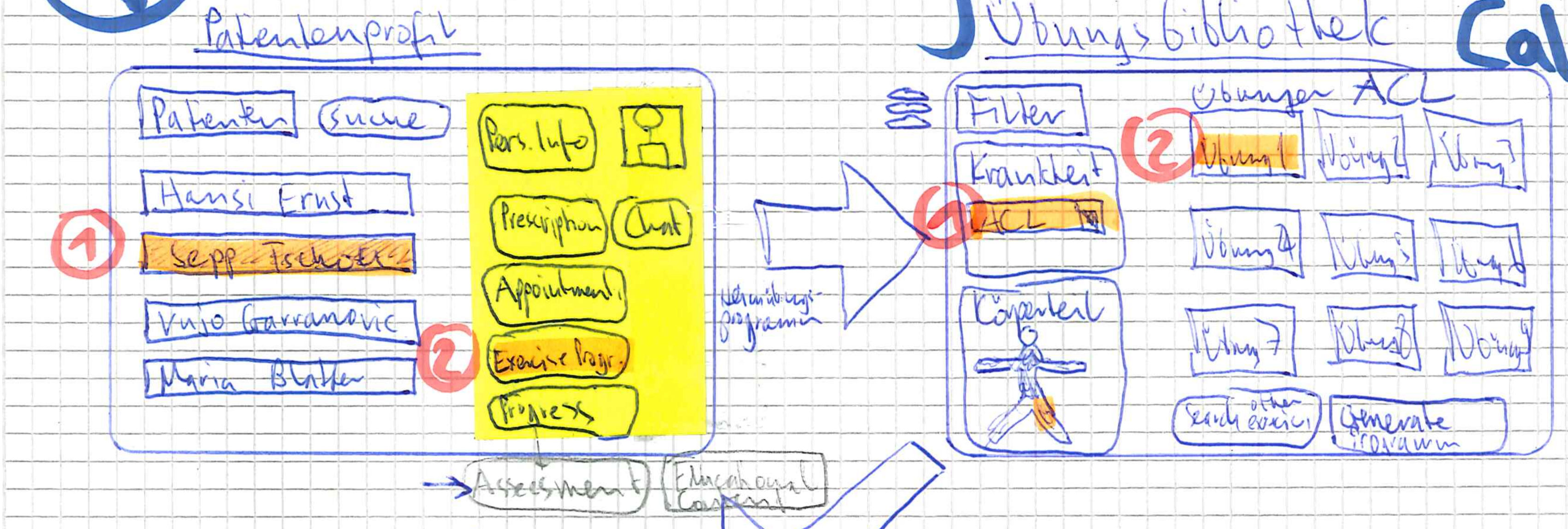

Übungseinstellung 1

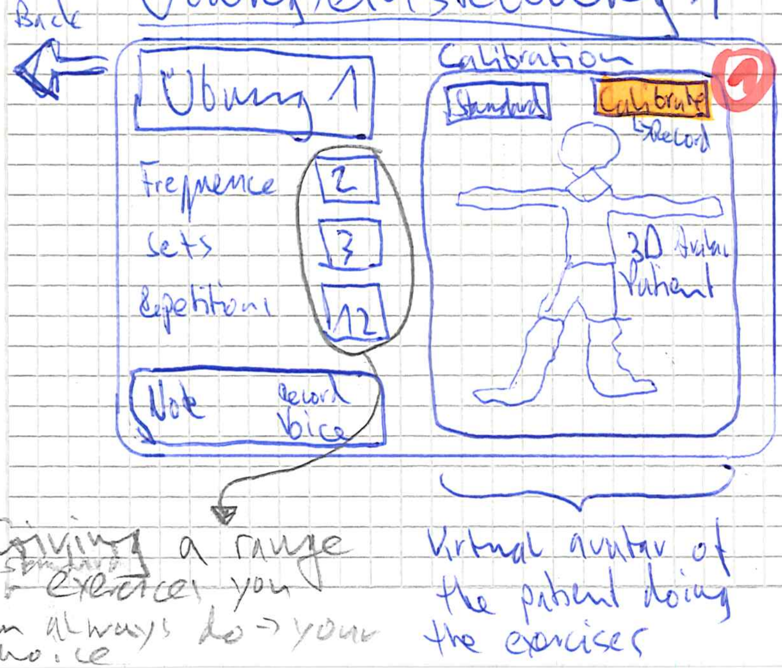

Übungseinstellung 2

- Patient wears glasses and performs exercise. If he/she performs it optimal to his/her physical capabilities the physiotherapist presses „RECORD"
- The optimal movement is saved in the Übungseinstellung 1

② Physio in-practice

Zu weisen von Questionnaires und Educational content

Generierung Übungsprogramm

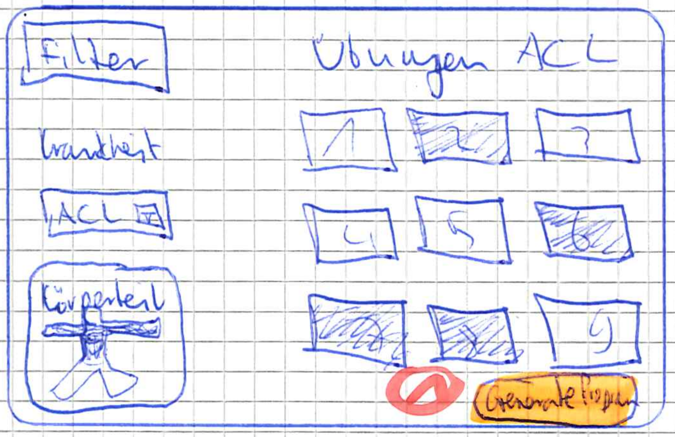

Weekly planner

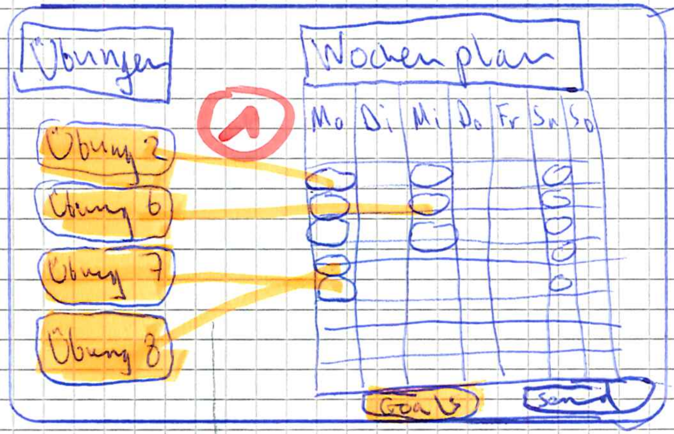

Alternativ
3 x pro Woche and
Wochenplan macht Patient selber

Drag and Drop oder automatisch

~~Tool~~ Tool instruction

- Physio therapist gives short instruction into the set up & use of the tool
- Gives the hardware to the patient

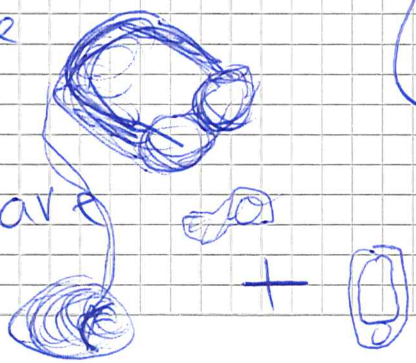

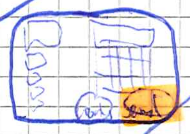

Quantitative
Qualitative → Maybe the digital physio coach should also use them to motivate the patients

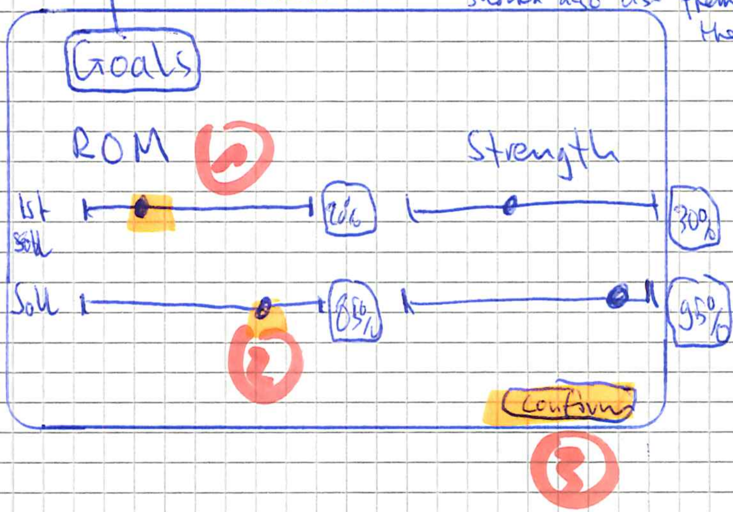

③ Patient @ home

Instruction

by conversational agent on mobile phone

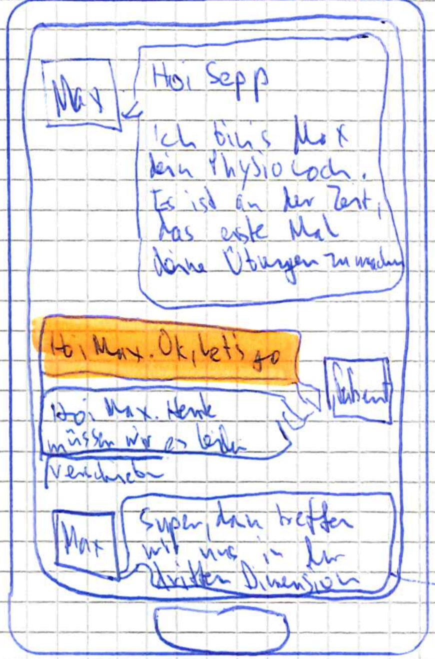

Bitte ziehe die Brille an, wie auf dem Video

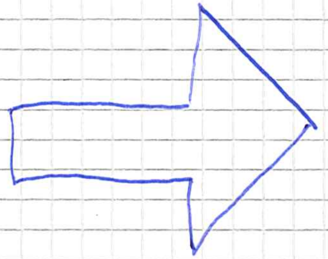

- Screen einfügen mit Educational Content
- Screen mit Erklärung von Max, wer er ist
- Screen mit Push notification
- Übung verschieben Screen

Performing the exercises

- Show Prototype
- Explain Vision
- + Patient gives Feedback
- + Patient adapts exercises (or does extra, voluntarily exercises)

④ Patient @ home

After exercises 1

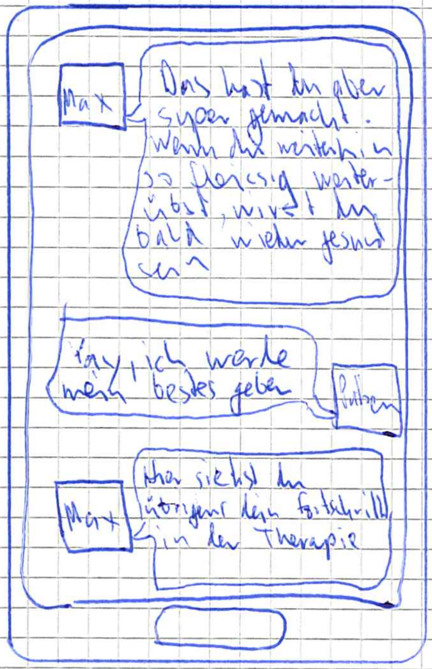

- Screen mit Therapiefortschritt
- Rewardsystem

After exercises 2 → Training abmachen

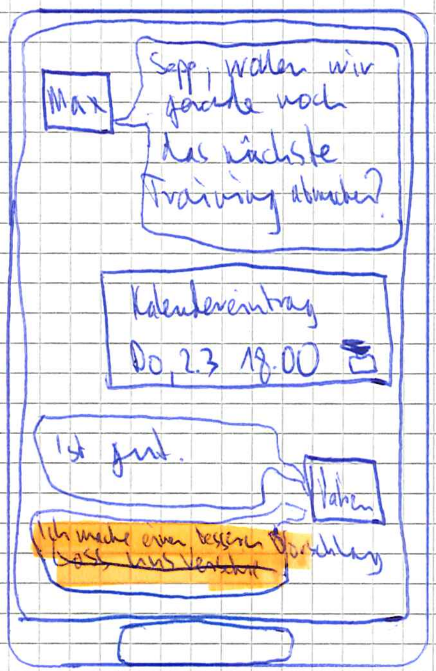

- Screen mit Kalendereintrags-änderung

(5) Patient between sessions

Patient has question

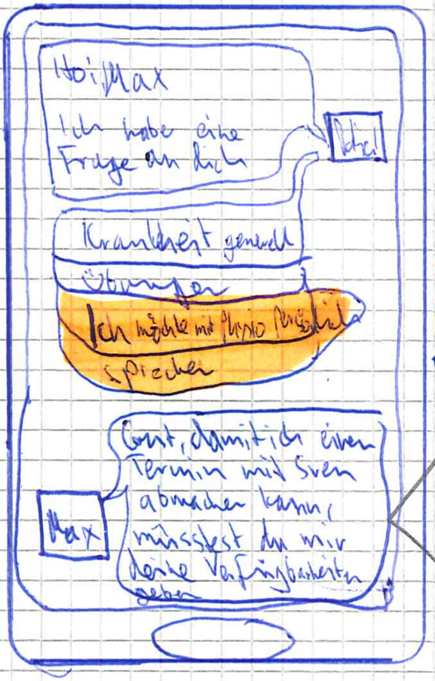

- Option: Patient demands new exercises
- Option: Patient receives Questionnaire

- Time lag

Kommunizieren

- Was hat Max für eine Rolle

▷ Chat im Triadgel?
↳ Not real-time

▷ Video Call
↳ Verfügbarkeit angeben

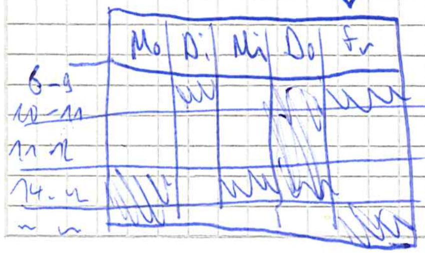

Patient receives motivation

by Max on the phone

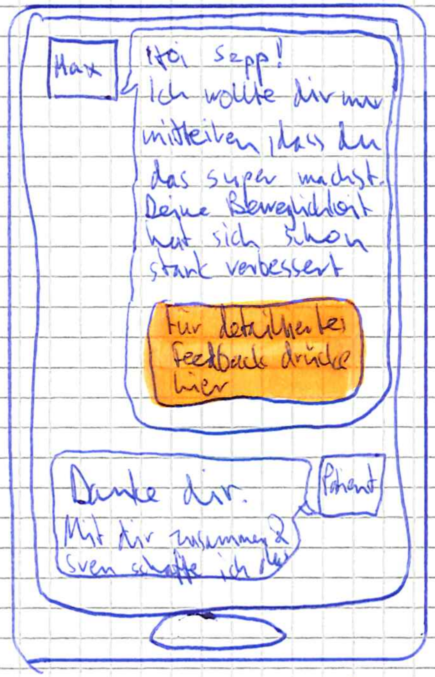

- Progress
- How are you?

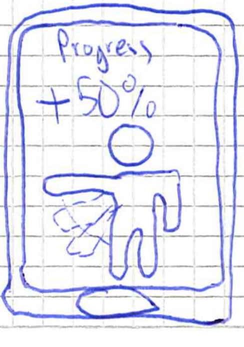

Detailliertes Feedback

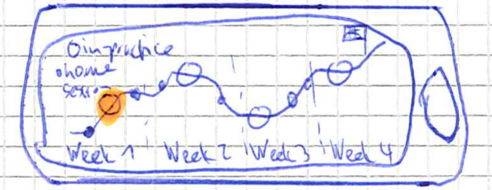

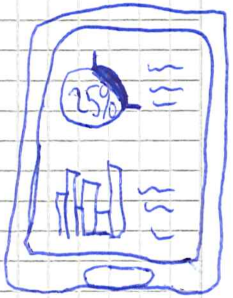

- Screen von einer Session

⑥ Patient between session

Patient gets reminded and prepared for next session

Home exercise session 2

• Home-Bildschirm

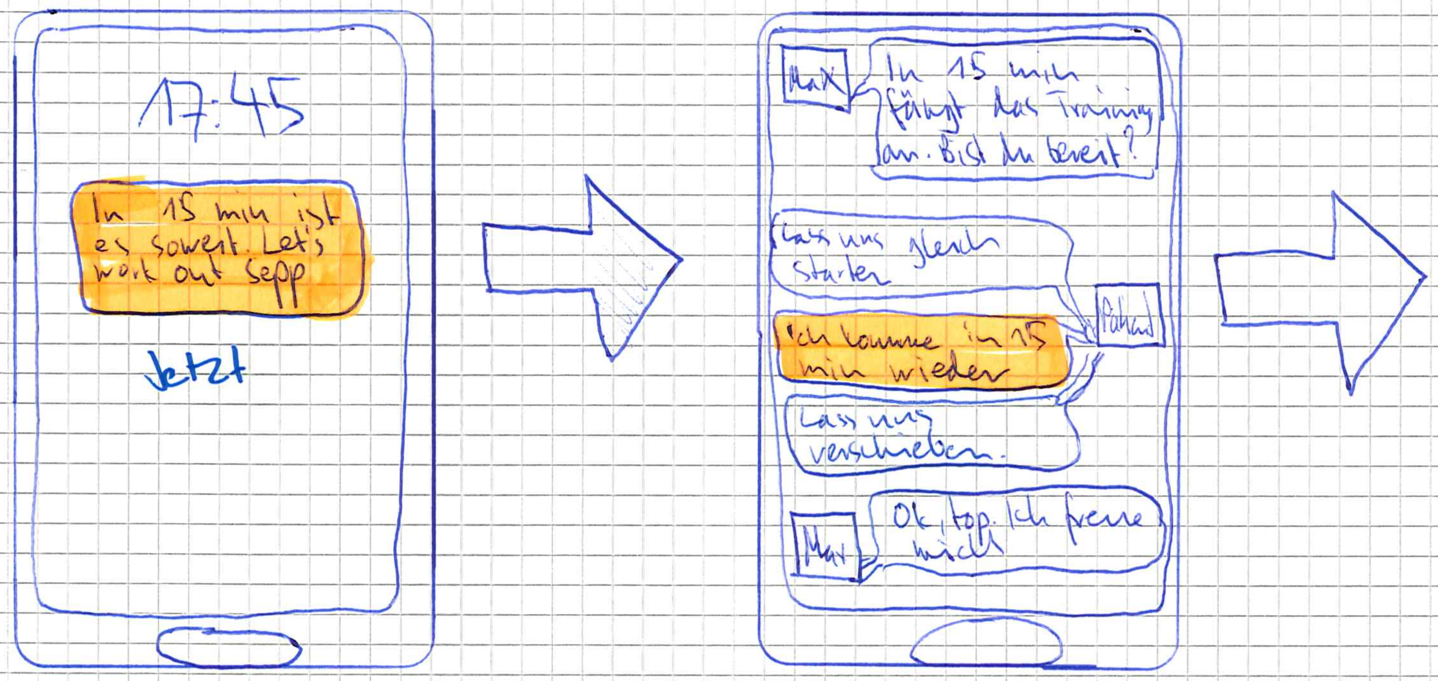

• Patient reports pain

7 Physio between sessions

Patient Monitoring

Patient Profile

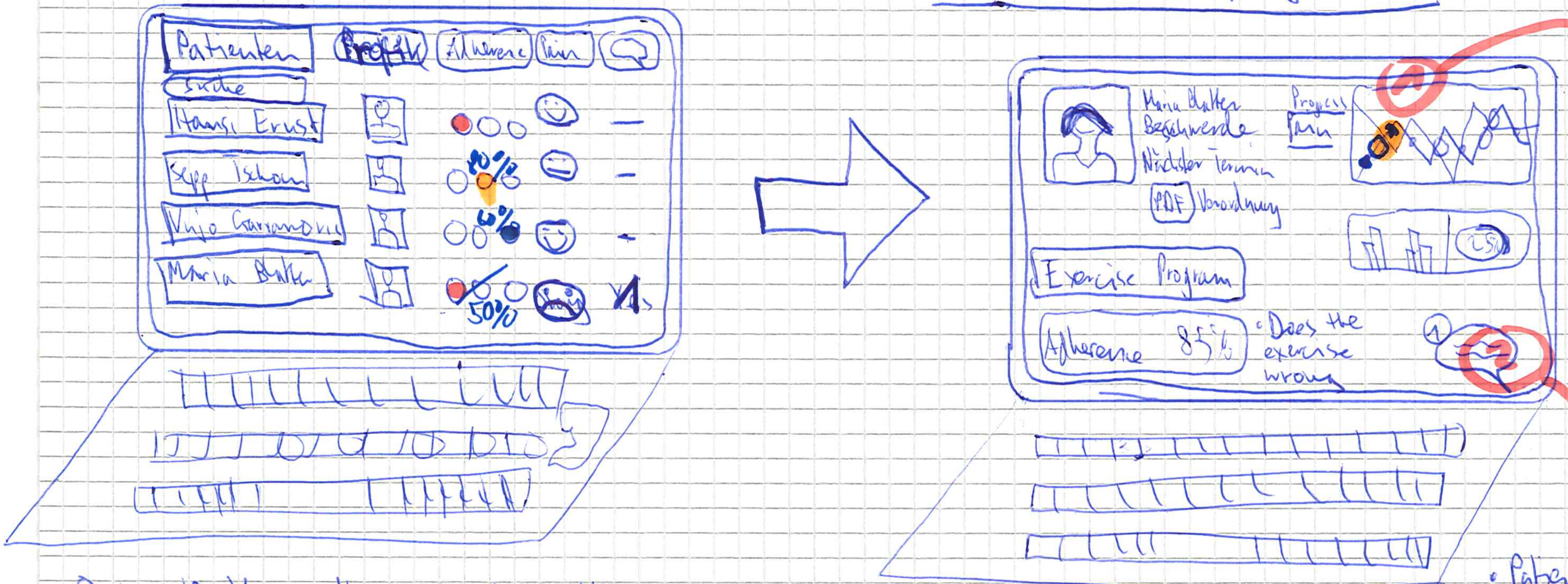

• Patientin hat bei der einen Übung in der Heimübung Schmerz angegeben

• Only if the adherence is really low, the patient has strong pain or if he request an online appointment, the physiotherapist receives a notification

• Patient hat eine persönliche Frage an den Physio

Diese Funktion kann nur als Selbstzahler genutzt werden

7.1 Physio between sessions

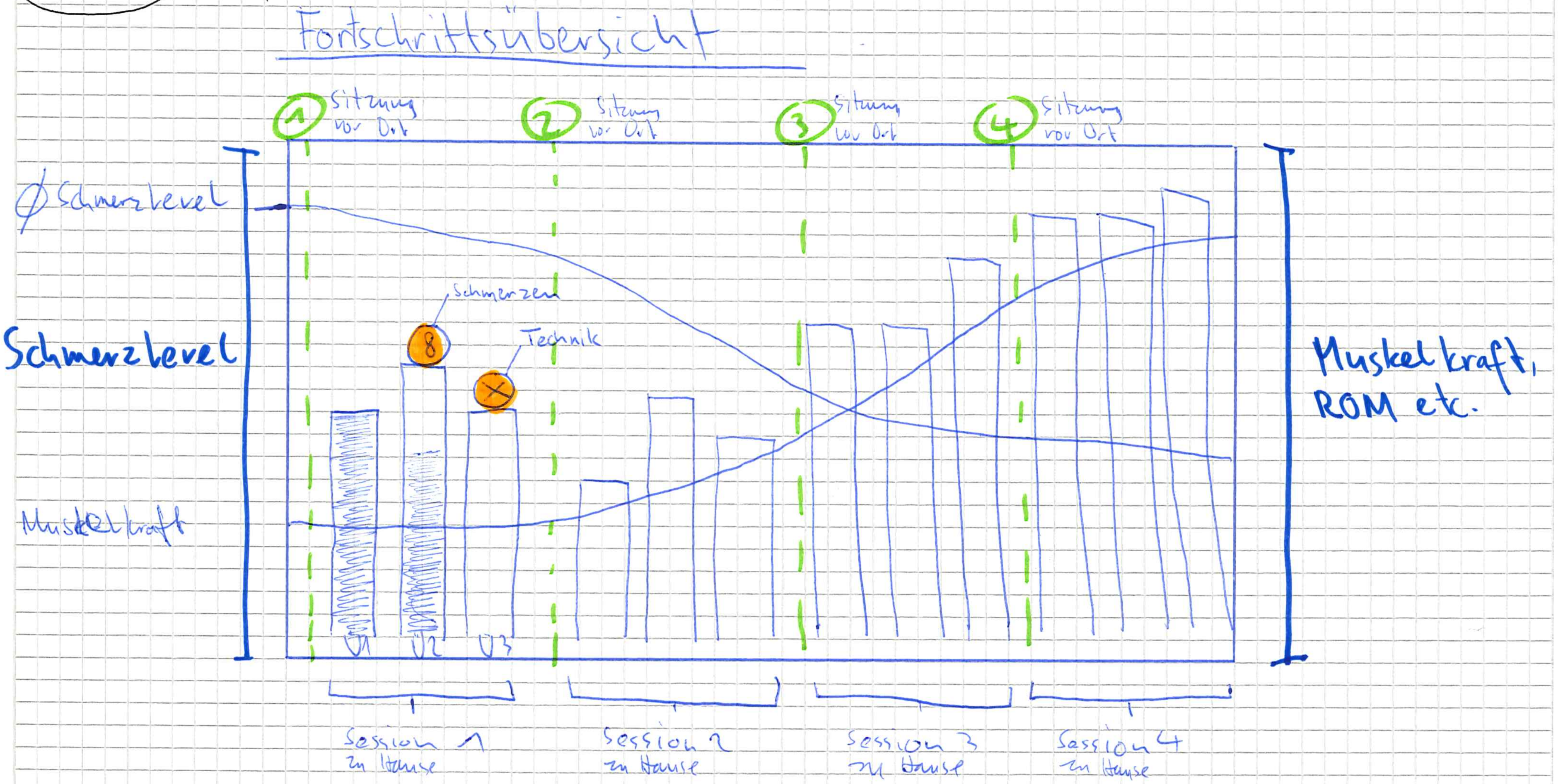

⑧ Physio between sessions

Patient reported pain on one exercise

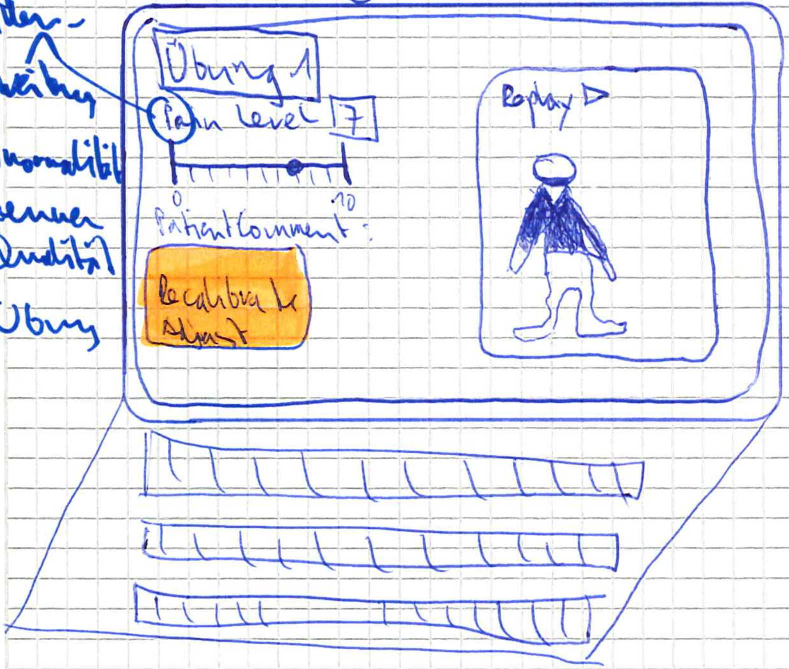

• Erkennt Schmerz aufgrund Pupillen-Ausweitung

• Abnormalität erkennen ↳ Qualität Übung

Remote therapy session with Patient

• Video call with patient

• New calibration of the exercise → Patient wears the glasses and does the exercise again

Remote adaption of program without patient

• Change the whole exercise

• Adapt single technique parameters

• Adapt frequency, sets, repetitions

⑨ 2nd in-practice session

Reminder to go to Physio

Push-notification

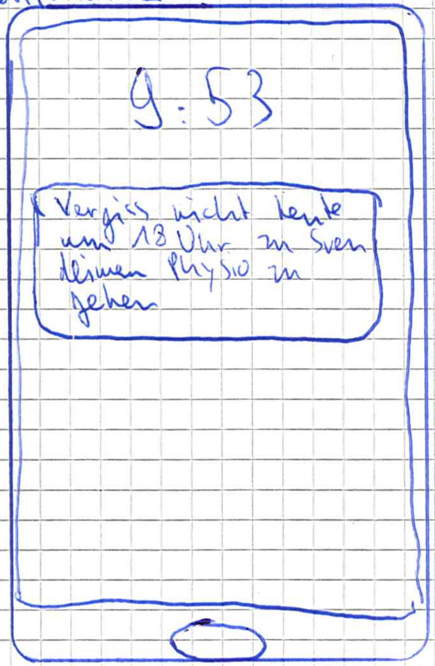

- Patient kann Physio sagen, was erreicht hat -> kann stolz darauf sein
- Als Diskussionsgrundlage
- Kunde zeigt, was er gemacht hat
- Physio weiss hat keine Zeit, um Sachen vorher einzusehen

- Zusätzlichen Screen zur Online Termin-vereinbarung

In-practice session

- Discuss the patient's progress and problems
  -> Quick summary and good visualizations needed in order to lose not too much time
- Test the patient's physical functions
  -> Determine to what extent the patient achieved his quantitative and qualitative goals
- Adapt the exercise program based on the precise feedback data and the in-practice assessement

Gesamtannahmen

① Patients pay for all in-between practice sessions on their own

② Remote sessions are reimbursed by the insurers

③ Target patients?

↳ Relatively tech-affine patients

↳ chronic patients
