# Supplementary material for: Hybrid Ubiquitous Coaching With a Novel Combination of Mobile and Holographic Conversational Agents Targeting Adherence to Home Exercises: Four Design and Evaluation Studies
Source: J Med Internet Res. 2021 Feb 22;23(2):e23612. doi: 10.2196/23612 (PMC7939948; doi:10.2196/23612)
Supplement: Multimedia Appendix 7 [file jmir_v23i2e23612_app7.docx]

# Multimedia Appendix 7: Questions of the semistructured interview of study 2

**English version (translated)**

**General**

- Do you think that through this digital coaching your patients could be better supported between physical therapy sessions with you?
- To what extent would the gained feedback on adherence and pain help you to optimize your therapy? Does such detailed feedback make sense to you at all? What other feedback data would be interesting for you? Show progress screen

**Patient-related variables**

- Would this digital assistant allow for more individualized coaching throughout the entire therapy cycle than has been possible up to now? Why?
- Do you think that Alex the digital physio coach can motivate your patients to consistently perform their home exercise programs more than methods you have used so far? Why? (MR Avatar, Chatbot)
- Should it be more playful?
- Do you think that through Alex the digital physio coach patients will have a more positive attitude towards their therapy and home exercise program? Why?
- What do you think about the way the system tries to provide patients with more information about therapy and the disease? Does this complement or expand your efforts to educate/train the patient in a positive sense?

**Frequency**

- How do you rate the Mobile Reminder function shown? Do you think that people will forget the exercises less / do more exercises than before?
- What do you think about the self-adjustable planning function? So the fact that the patient is encouraged to plan his training (independently or together with you), but always has the freedom to adjust the planning (even at short notice)?

**Sets and repetitions**

- How do you like the fact that the patient is guided interactively through the home exercise program by Alex the physio coach? How do they rate the added value compared to the methods they use?
- Does this better ensure that patients perform the prescribed sets and repetitions during home exercise programs?

**Technology**

- How do you assess the added value of this additional spatial dimension to learning movements compared to exercise sheets with pictures/stroke men + explanations and video instructions. And why?
- How do you estimate the added value of this kind of real-time feedback on the execution of movements?
  - Prototype status now?
  - Exact feedback? How exact?
- How individual should the feedback be? Show personalization of the exercise
- What do they find better: If the patient receives feedback during the exercise by means of a virtual image of himself or the avatar of the physio coach?

**Working Alliance**

- Do you think that the digital assistant could help you to develop a common understanding of the therapeutic goals? (Communication and clarification of the goals even in the absence of the physio)
- Do you think that the digital assistant could help you to develop a common understanding of how to carry out the exercises? In other words, help you to ensure that the exercises are carried out correctly in the patient's home, even when you are not on site)
- Do you think this system could help to strengthen the trust between you and your patients? (support between physical sessions, more security)

**Intention to use and integration into the a patient’s journey**

- Could you imagine using this system as described above in the therapy of your patients in the future? Do you see an added value to the status quo? Why?
- How do you estimate the relation between additional expenditure and added value of this system?
- In your opinion, what are the reasons why the described embedding in the patient pathway could fail from a process-related perspective?
- In your opinion, where is the greatest potential for improvement?
- How do you find the triage of the questions?
- Could you imagine conducting two shortened remote therapy sessions instead of one physical therapy session?
- What are the most positive and negative aspects of digital physio coaching that come to your mind spontaneously?

**German version (original version)**

**Allgemein**

- Denken sie, dass durch dieses digitale Coaching Ihre Patienten besser zwischen den physischen Therapiesitzungen mit Ihnen unterstützt werden könnten?
- Inwiefern würde Ihnen das gewonnen Feedback zur Adhärenz und zum Schmerz helfen, ihre Therapie zu optimieren? Macht ein solch detailliertes Feedback für Sie überhaupt Sinn? Welche weiteren Feedbackdaten wären für sie interessant? *Zeige Fortschrittsscreen*

**Patientenbezogene Faktoren**

- Würde dieser digitale Assistent ein individuelleres Coaching über den gesamten Therapiezyklus hinweg ermöglichen als bisher? Warum?
- Denken Sie, dass Alex der digitale Physiocoach Ihre Patienten mehr dazu motivieren kann, ihre Heimübungsprogramme konsequent durchzuführen als bisherig von Ihnen eingesetzte Methoden? Warum? *(MR Avatar*, *Chatbot)*
  - Sollte es noch spielerischer sein?
- Denken Sie, dass durch Alex der digitale Physiocoach die Patienten eine positivere Einstellung gegenüber ihrer Therapie und ihre Heimübungsprogramm erhalten? Warum?
- Wie finden Sie die Art und Weise, wie das System versucht, den Patienten weiterführende Informationen zur Therapie und zur Krankheit zu vermitteln? Ergänzt oder erweitert dies Ihre Bemühungen, den Patienten zu erziehen/bilden, im positiven Sinne?

**Frequenz**

- Wie beurteilen sie die gezeigte Mobile Reminder Funktion? Denken sie, dass die Leute dadurch die Übungen weniger vergessen/ mehr machen als bisher?
- Was denken sie über die selber anpassbare Planungsfunktion? Also dem Fakt, dass der Patient zwar dazu animiert wird, sein Training zu planen (selbständig oder mit Ihnen zusammen), jedoch stets die Freiheit hat, die Planung (auch kurzfristig) anzupassen?

**Sets und Repetitionen**

- Wie finden sie es, dass der Patient von Alex dem Physiocoach interaktiv durch das Heimübungsprogramm geführt wird? Wie beurteilen sie den Mehrwert im Vergleich zu denen von ihnen eingesetzten Methoden?
- Wird so besser sichergestellt, dass die Patienten die verschriebenen Sets und Repetitionen bei Heimübungsprogrammen durchzuführen?

**Technik**

- Wie beurteilen Sie den Mehrwert dieser zusätzlichen räumlichen Dimension auf das Lernen von Bewegungen im Vergleich zu Übungsblättern mit Bildern/Strichmännchen + Erklärungen und Videoinstruktionen. Und warum?
- Wie schätzen Sie den Mehrwert dieser Art von real-time Feedback bezüglich der Bewegungsausführung ein?
  - Prototyp Stand jetzt?
  - Exaktes Feedback? Wie exakt?
- Wie individuell müsste das Feedback sein? *Zeige Personalisierung der Übung*
- Was finden sie besser: Wenn der Patient während der Übungsdurchführung Feedback anhand eines virtuelles Abbildes von sich selbst oder dem Avatar des Physiocoaches erhält?

**Zusammenarbeit**

- Denken Sie, dass der digitale Assistent Sie dabei unterstützen könnte, ein gemeinsames Verständnis zu den Therapiezielen zu entwickeln? (*Kommunikation und Verdeutlichung der Ziele auch in Abwesenheit des Physios)*
- Denken Sie, dass der digitale Assistent Sie dabei unterstützen könnte, ein gemeinsames Verständnis zur konkreten Übungsdurchführung zu entwickeln? *Also Sie dabei unterstützt, die richtige Übungsdurchführung beim Patienten sicherzustellen, auch wenn Sie nicht vor Ort sind)*
- Denken Sie, dass dieses System dabei helfen könnte, das Vertrauensverhältnis zwischen Ihnen und Ihren Patienten zu stärken? *(Unterstützung zwischen den physischen Sitzungen, mehr Sicherheit)*

**Nutzungsabsichten & Einbettung in den Behandlungspfad**

- Könnten Sie sich vorstellen, dieses System wie es beschrieben wurde, zukünftig in der Therapie bei ihren Patienten einzusetzen? Sehen sie einen Mehrwert zum Status-quo? Warum?
  - Wie schätzen sie das Verhältnis zwischen Mehraufwand und Mehrwert dieses Systems ein?
- Was sind Ihrer Meinung nach Gründe, woran die beschriebene Einbettung in den Patientenpfad aus prozessualer Sicht scheitern könnte?
  - Wo hat besteht Ihrer Meinung nach das grösste Verbesserungspotential?
  - Wie findet ihr die Triage bei den Fragen?
- Könnten Sie sich vorstellen, anstelle von einer physischen Therapiesitzung zwei verkürzte remote Therapiesitzungen durchzuführen?
- Was sind die positivsten und negativsten Aspekte des digitalen Physiocoaches, die Ihnen spontan in den Sinn kommen?
