# Supplementary material for: Hybrid Ubiquitous Coaching With a Novel Combination of Mobile and Holographic Conversational Agents Targeting Adherence to Home Exercises: Four Design and Evaluation Studies
Source: J Med Internet Res. 2021 Feb 22;23(2):e23612. doi: 10.2196/23612 (PMC7939948; doi:10.2196/23612)
Supplement: Multimedia Appendix 10 [file jmir_v23i2e23612_app10.docx]

# Multimedia Appendix 10: Questions of the semistructured interview of study 3

**English version (translated)**

Introduction: "This part is about finding out how you experienced the different instructions and how they address the problems we discussed before. We are also interested in how the Physio-Coach can be improved to better address the problems.

- How did you experience the Video/Paper/Physio-Coach instruction?
- Which one did you like best and why?
- How do you think the Video/Paper/Physio-Coach instruction addresses the problems discussed earlier?
- Do you have any ideas or suggestions how to improve the Physio-Coach?

**German version (original version)**

Einleitung: ”In diesem Teil geht es darum herauszufinden, wie sie die verschiedenen Instruktionen erlebt haben und wie diese die Probleme welche wir vorher diskutierten adressieren. Ausserdem interessiert uns wie der Physio-Coach verbessert werden kann um die Probleme besser zu adressieren.”

1. Wie haben Sie die Video/Papier/Physio-Coach Instruktion erlebt?
2. Welche hat Ihnen am Besten gefallen und wieso?
3. Wie denken Sie adressieren die Video/Papier/Physio-Coach Instruktion die vorher besprochenen Probleme?
4. Haben sie Ideen oder Vorschläge wie der Physio-Coach verbessert werden könnte?
