# Supplementary material for: Hybrid Ubiquitous Coaching With a Novel Combination of Mobile and Holographic Conversational Agents Targeting Adherence to Home Exercises: Four Design and Evaluation Studies
Source: J Med Internet Res. 2021 Feb 22;23(2):e23612. doi: 10.2196/23612 (PMC7939948; doi:10.2196/23612)
Supplement: Multimedia Appendix 12 [file jmir_v23i2e23612_app12.docx]

# Multimedia Appendix 12: Examples of the smartphone-based conversational turns of study 4

To maintain the privacy of the patient, the names in the conversations are fictitious. The intervention messages were originally in German. Conversational examples for reminders, psychoeducational material and feedback were translated into English and included. It is important to note that the messages were literally translated, thereby they might not have the same effect or meaning as the original German ones.


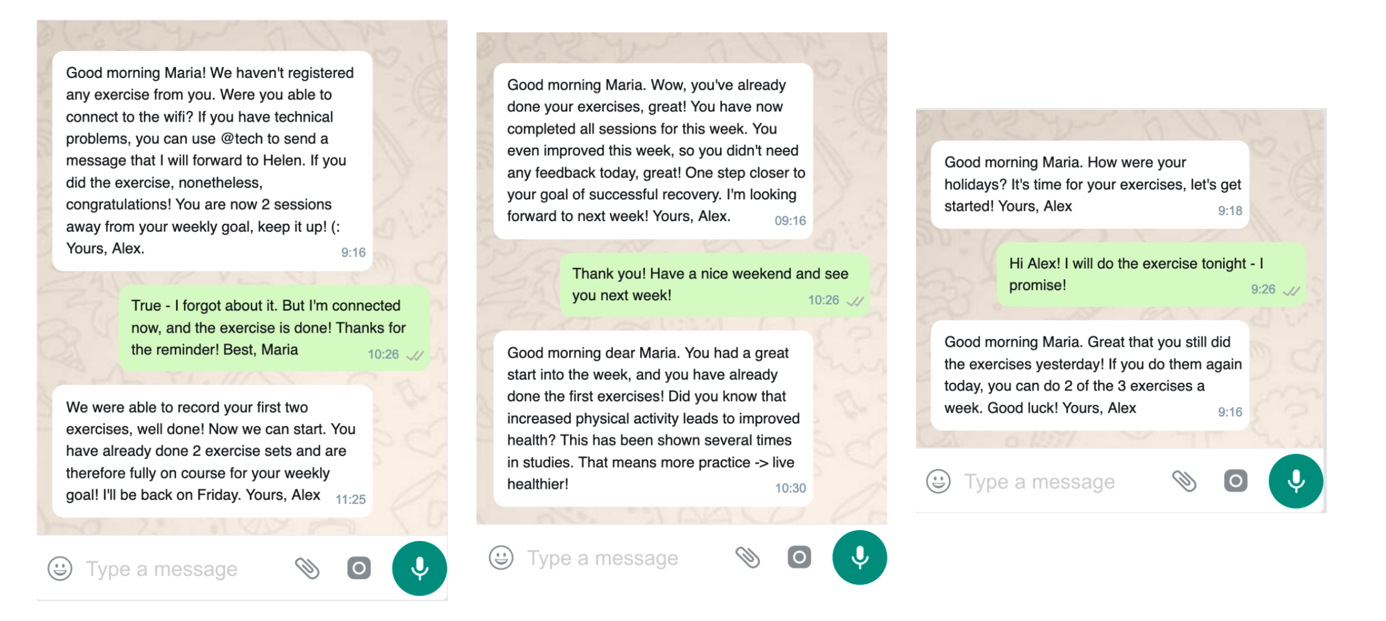


Figure 1: Conversational turn with a) reminder messages (left), b) psycho-educational material (center), c) motivational messages (right).


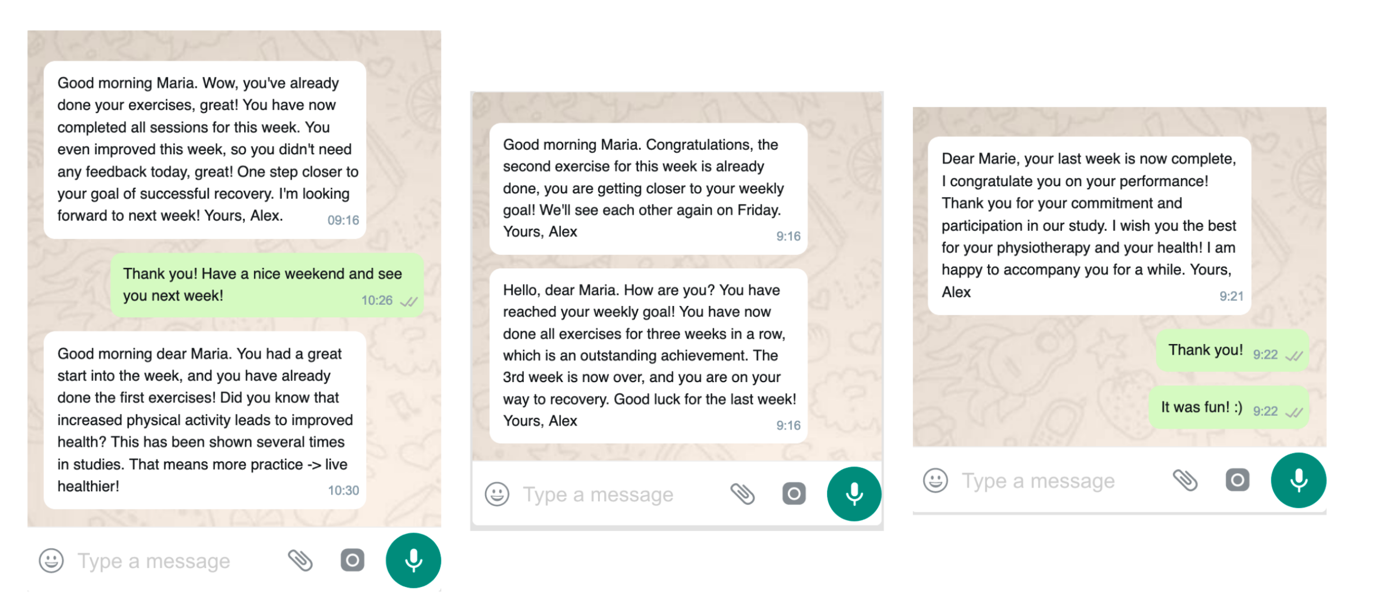


Figure 2: Conversational turn including progress report
